# First Reports of Effects of Insulin, Human-like Insulin Receptors and Adapter Proteins in *Acanthamoeba castellanii*

**DOI:** 10.1038/s41598-020-63435-4

**Published:** 2020-07-16

**Authors:** Abdul Mannan Baig, Areeba Khaleeq

**Affiliations:** Department of Biological and Biomedical Sciences, Aga Khan University, Karachi, Pakistan

**Keywords:** Parasitology, Parasite biology, Evolutionary genetics, Evolutionary developmental biology, Cell biology, Computational biology and bioinformatics, Evolution, Microbiology

## Abstract

The insulin receptor (IR) and insulin-like growth factor 1 receptor (IGF1-R) play key roles in growth, regulation of nutrient metabolism and carbohydrate homeostasis. Insulin-like molecules in prokaryotes and other early life have been reported. However, an account of metabolic effects of insulin, transcriptomic evidence of expression of glucose transporting channels (GLUT) and homology modelling of IR and IGF1-R like proteins in unicellular life-forms have yet to be established. *Acanthamoeba* spp. has existed for about 2 billion years and is one of the earliest mitochondriate unicellular eukaryotic cells on Earth. Despite *Acanthamoeba* spp. being grown in a medium called peptone-yeast-glucose (PYG) for over 50 years, the mechanism and regulation of glucose uptake by IR or IGF1-R molecules in this microbe has not yet been reported. Several methods were utilized to validate the effects of insulin on trophozoites of *A. castellanii*, including: growth assays with insulin, estimation of glucose and potassium (K^+^) entry into the cell, and histology showing anabolic effects on proteins. Bioinformatic computational tools and homology modeling demonstrated the involvement of IR like proteins, GLUT, and adapter proteins in mediating the IR cascade. Growth assays showed proliferative effects in a dose range of 2.98–5.97 µmol/mL of insulin. After insulin exposure, *A. castellanii* trophozoites displayed enhanced Periodic acid-Sciff (PAS) staining. Amino acid sequence similarities and homology modelling revealed ACA1_163470 in *Acanthamoeba* spp. to be a homolog of human-IR. *Acanthamoeba* protein ACA1_336150 shares similarities with IGF1-R. Additionally, some proteins like ACA1_060920 have attributes of GLUT like channels on homology modelling and show similarity with human GLUT. Knowledge of IR and insulin effects in *Acanthamoeba* spp. contributes to its biology and advances current understanding behind the evolution of IR and IGF1-R signalling cascade.

## Introduction

Unicellular and multicellular species that exist on Earth utilize glucose as a source of energy. Primitive species such as prokaryotes, as well as evolutionarily advanced species, like humans, metabolize glucose to produce adenosine triphosphate (ATP) to use as an energy source. The selection of glucose as a preferential nutrient for obtaining energy is possibly its abundance in nature. In the last decade, research in the field of comparative biology has increased exponentially. Scientists worldwide are tracing the evolutionary origins of proteins and receptors like the insulin superfamily, insulin receptor (IR), insulin-related peptides (IRP), and the insulin-like growth factors (IGF). Adapter proteins related to glucose homeostasis are present in humans but have also been found in some primitive unicellular organisms. In the 1980s, glucose and its regulators such as insulin-like molecules were reported in protozoa (*Tetrahymena*), fungi (*Aspergillus fumigatus* and *Neurospora crassa*), and prokaryotes^1,2^. After discovering insulin-like molecules in the pre-genomic era, scientists postulated that additional research would determine the sequence of genes derived from unicellular organisms and reveal the ancestral origin of insulin and IR like proteins in these early life-forms. Other studies of insulin and insulin-like molecules, suggest that insulin could have taken its origin from conserved insulin genes in unicellular organisms^3^. The identification of a precursor pre-proinsulin-like pseudogene from the *N. crassa* genome by PCR^4^ supports the molecular evolutionary hypothesis. Later studies found molecules that produce metabolic effects in mammalian adipocytes resembling human insulin in *N. crassa*^5^. Additionally, a purified membrane-bound insulin binding protein in *N. crassa* cells has been suggested to be a “receptor” that mediates downstream metabolic effects of insulin^6^. Insulin precursor expression and secretion, including proinsulin, has also been reported in *S. cerevisiae*^7^. Insulin binding sites in *Amoeba proteus* have previously been described. However, evidence of insulin receptor (IR), and elucidation of GLUTs like glucose transporting channels using bioinformatics computational tools and homology modeling have not yet been reported in microbial pathogens or eukaryotic free-living amoeba (FLA). FLAs (like *Acanthamoeba* spp. and *Naegleria fowleri*) are known to grow in media that includes glucose as a basic nutrient for growth and proliferation^8,9^. Nelson’s culture medium consisting of 1 g liver digest and glucose per L of Page saline, supplemented with 2% donor calf serum has been used to cultivate *Naegleria fowleri*^8^. PYG medium is also rich in glucose, and has been used for the growth of the trophozoite forms of *Acanthamoeba* spp. for over 5 decades^9–12^. *Acanthamoeba* spp. consume glucose and execute carbohydrate metabolic pathways like glycolysis and gluconeogenesis^13^. However, no reports currently exist elucidating the mechanisms involved in glucose uptake into the cytosol, expression of GLUT-like proteins involved in glucose uptake, or expression of IR-like cell surface proteins in this organism. With the recent availability of the genome of FLAs in databases like AmoebaDB^14^, UniProtKB and NCBI^15,16^, investigating the above-mentioned adapter proteins involved in glucose homeostasis has become possible. These databases can be further leveraged to search for proteins like human IR, IRP, IGF and GLUT transporter as “model molecules” to find their homologs in *Acanthamoeba*. Using subsequent bioinformatics computational tools, the candidate proteins returning as matches can be subjected to further in-depth analysis by homology modeling and ligand docking prediction in order to determine their structural details and similarity with human glucose homeostasis regulating proteins. *Acanthamoeba* spp. has been used as a model organism to study complex eukaryotic physiological processes like the role of voltage-gated calcium channels (VGCC) in the regulation of cellular proliferation and growth^17^ and the proteins involved in actin dynamics, glycolysis, and proteolysis^18^. This unicellular microbe has been studied to understand the origin of G-protein coupled receptors^19^, muscarinic receptor-like proteins^20^ and signal transduction pathways activated by Acetylcholine (ACh) in non-neuronal cells. Glucose utilization and metabolism via aerobic and anaerobic glycolysis have been reported^13,21^, but the processes involved in glucose uptake and adapter molecules regulating its metabolism remain to be elucidated. Selecting *Acanthamoeba castellanii* (belonging to the T4 genotype) as a model unicellular eukaryote we intended to show evidence of target human-like IR and GLUT like proteins and experimental confirmation of the effects of insulin in this protest pathogen. Determination of effects of insulin and showing evidence of IR and IRS like proteins in this unicellular eukaryote is expected to broaden our understanding of the commonalities and differences in metabolic handling of glucose by unicellular eukaryotes and humans. This knowledge could have far-reaching implications possibly helping to better understand the complex metabolic handling of glucose by dysregulated eukaryotic entities like cancer cells. Previously the metabolic processes involved in encystation in *Acanthamoeba* spp. has been studied and compared with the cancer dormancy^22^, a metabolically quiescent state of malignant cells, from which they revert (as is true for cyst turning into trophozoites) to an active state to cause recurrences months to years after chemotherapy. *Acanthamoeba* spp. can cause significant morbidity and mortality, including: vision-threatening *Acanthamoeba* keratitis (AK), wound infections and granulomatous amoebic encephalitis (GAE), which is almost always fatal^9–12^. Understanding the differences in adapter proteins involved in glucose homeostasis between human and *Acanthamoeba castellanii* could also enable the synthesis of glucose metabolic silencing molecules to target and kill this protist pathogen in AK and GAE without affecting human proteins involved in glucose homeostasis regulation.

## Materials and Methods

### Drugs, chemicals, analyzers and dyes

Short-acting insulin (ACTRAPID) was purchased from Novo Nordisk. 100 IU/mL solution for injection in the vial was diluted to prepare a stock solution. Metformin hydrochloride was purchased from Sigma-Aldrich. Hematoxylin and Eosin (H&E) staining were done at Microscopy labs at Aga Khan University. Fura-2AM (LOT# 1874816) was purchased from Invitrogen -Thermofisher Scientifics for calcium imaging. For glucose estimation, catalogue #- GL2614 - Randox Labs kit was used that determines glucose by GOD-PAP method. An automated analyzer used in our labs that measures serum K^**+**^ in humans was employed for K^+^ estimation in mEq/L in PYG incubated with *A. castellanii* trophozoites as control and compared with K^+^ levels in the medium of insulin-treated cells.

### Cell cultures

#### Acanthamoeba cultures

In our lab, keratitis isolates of *A. castellanii* trophozoites (cellular active forms) belonging to the T4 genotype are routinely grown in 10 ml of PYG medium (0.75% [wt./vol] proteose peptone, 0.75% [wt./vol] yeast extract, and 1.5% [wt./vol] glucose) in T-75 tissue culture flasks. They are kept at 37 °C without shaking for 24 h and used in experiments, as has been done in our previous experiments^20^. The PYG media was refreshed 15 to 20 h prior to experiments. *A. castellanii* cells adhered to the floor of the flasks represent the healthy trophozoite forms and were collected by placing the flasks on ice for 30 min followed by gentle agitation. A variable number of the trophozoites, growth medium and insulin concentrations were selected for different experiments. Growth medium PG (Peptone-glucose) without yeast was selected for testing short term effects of insulin at 15, 30 and 60 mins.

#### Growth and cytotoxic assays

Growth assays were done to determine the effects of insulin on the proliferation of the cells. *A*. *castellanii* trophozoites (0.5 × 10^**6**^ amoebae/mL/well) were incubated in PYG medium with 2.98 and 5.97 µmol/mL of insulin alone and insulin plus metformin 100 µg/mL in 24-well plates at 30 °C for 48 h. After the incubation, the amoebae were counted using a hemocytometer. The data are represented as the means and standard errors of at least three independent experiments performed in duplicate. To determine the toxic effects of insulin in *A. castellanii*, briefly, *A. castellanii* trophozoites from an entire flask (~12 million) were incubated with 15.53–29.8 µmol/mL of insulin in PYG in 6-well plates. The plates were incubated at 30 °C for 24 h. Following this incubation, amoeba viability was determined by adding 0.1% trypan blue and determining the number of live (non-stained) and dead (stained) cells using a hemocytometer. The counts from *A. castellanii* incubated with PYG alone (without insulin) were used as controls. Data are represented as the means and standard errors of at least three independent experiments performed in duplicate.

### Periodic acid–Schiff (PAS) staining of *Acanthamoeba castellanii*

The effects of insulin on human cells includes glucose influx followed by glycogen synthesis and storage. In order to evidence, a similar effect in trophozoites of *Acanthamoeba castellanii* PAS staining was performed. Healthy trophozoites were harvested from the wells and collected in separate tubes and centrifuged at 2500 rpm for 10 minutes. The supernatant was discarded and the pellet was washed with sterile PBS. The pallet was resuspended in 4% formaldehyde for 10 minutes. The pallet was centrifuged again for 5 min at 2500 rpm and the supernatant was discarded. The cells were spread over the glass slide and allowed to dry. The slides were washed with distilled water to rehydrate them. Fixed cells on slides were exposed to 0.5% PAS solution for 5 minutes. The slides were washed again with distilled water for 5 mins. Schiff reagent was added and left for 15 minutes. Slides were washed in lukewarm water for 5 minutes and were left to get dry. All the slides were counterstained with hematoxylin for 1 minute and slides were washed again in distilled water for 5 minutes. A drop of the hydrophilic mounting medium was added to the slides and coverslips were placed on it and allowed to dry. Slides were observed under Olympus BX41 microscope (Center Valley, PA) and images at 40x were captured.

### Imaging of insulin and metformin-treated trophozoites

The *Acanthamoeba castellanii* trophozoites were exposed to 2.98–15.53 µmol/mL of insulin, 100 µg/mL of metformin alone and with 100 µg/mL of metformin in combination with 15.53 µmol/mL of insulin for 24 h. Images (40×) were taken using vista vision Olympus inverted microscope, immediately before incubation and after 24 h to observe the changes in cellular morphology and trophozoite count per high power field.

### Determination of Insulin effects on Glucose and Potassium entry inside the cells

*Acanthamoeba* trophozoites grown in PYG (12 × 10^6^) were tested for glucose entry and potassium K^+^ influx induced by insulin. Concentrations of glucose and K^+^ were measured in the control and incubations with insulin. A non-toxic medium that mimics human plasma, like PBS and Peptone/Glucose (PG) without 2-deoxyglucose was prepared to test the effects of insulin on glucose uptake in trophozoites before and after exposure to this hormone. Measurements of glucose concentrations were done at 15, 30 and 60 mins and 24 h in controls and incubations of trophozoites with insulin to observe the short- and long-term effects of this hormone on glucose uptake. For glucose estimation catalogue #- GL2614 of Randox Labs kit was used that determines glucose by GOD-PAP method. An automated analyzer used in our labs to measure K^+^-levels in human plasma was employed for K^**+**^ estimation in mmol/L in control wells (with trophozoites in PYG without insulin) and wells with trophozoites exposed to insulin. The trophozoites were harvested from the flask and centrifuged at 2500 rpm for 10 minutes and the pellet was resuspended in PBS to count the cells. About 12 million trophozoites were seeded in different wells plates in PYG (in duplicate). Insulin was immediately added to the wells that were incubated with trophozoites and the same volume of PBS was added in the control wells. The well plates were kept at room temperature and the supernatant was collected after 60 mins and centrifuged to remove any remaining cells in the supernatant in all of the wells and stored at −80 °C. The samples were sent to the lab to test the potassium levels.

### Hematoxylin and Eosin (H&E) staining of *Acanthamoeba* trophozoites

*Acanthamoeba* trophozoites 1 × 10^**6**^ were grown on coverslips in six-well plates within the PYG medium and treated with insulin alone and insulin plus metformin. After 24 hours of the treatment, media was removed from all the wells plates and cells (adhered in wells) were washed twice with PBS. The trophozoites on coverslips were exposed to hematoxylin stain for 30 seconds and washed off with PBS thrice. Cells were dehydrated by adding 95% alcohol and then exposed to Eosin for 10–15 seconds. This method was repeated with 100% alcohol which was removed immediately. Coverslip containing H&E treated cells were mounted with DPX mounting medium. The slides were allowed to dry for an hour and observed under a microscope.

### Calcium influx induction by insulin: Ca^2+^ staining by Fura-2AM

For intracellular calcium imaging in *Acanthamoeba* trophozoites a fluorescent molecular probe, Fura-2/AM was used. *Acanthamoeba* trophozoites 1 × 10^6^ were seeded in six-well plates along with the complete medium. 2 mL of ringer’s lactate solution was added additionally to provide free Ca^**2+**^ in media. Trophozoites were then exposed to the insulin and the plate was incubated for an hour at room temperature. After the treatment, the supernatant was discarded and cells were washed twice with phosphate buffer saline. Trophozoites were detached and moved to the Eppendorf tubes and centrifuged for 5 minutes at 2500 rpm, RT. A working solution of 5 µM Fura-2 AM was prepared and cells were suspended in and incubated with Fura-2/AM for an hour at room temperature in the dark as described previously^19^. The trophozoites were centrifuged and the pellet was washed with PBS twice and re-suspended in the fluorescent mounting medium. Cells were then transferred on the glass slide and coverslip was applied. After an hour the slides were observed under a fluorescent microscope and images of stained cells were captured at 40x magnification.

### Bioinformatics approach and computational tools

The proteins involved in glucose homeostasis and uptake in humans that are well established for their amino acid sequence and structures were taken as candidate molecules to search for homolog proteins in genome databases of *Acanthamoeba castellanii* T4 genotype. The AmoebaDB.org^14^, NCBI^16^ and UniProtKB^15^ were used to retrieve data of *Acanthamoeba* proteins that appeared to be the best match of the human protein submitted as a query. The Basic Local Alignment Search Tool (BLASTp) was used for identification of a protein homologs in *Acanthamoeba* spp. and determination of sequence similarities between human IR, IGF1-R, GLUT and other adapter *A. castellanii* proteins involved in IR signalling.

#### Amino acid sequence similarity between human and *Acanthamoeba* proteins involved in glucose homeostasis

The amino acid sequence of both human and *A. castellanii* proteins were aligned alongside each another to determine the percentage of sequence identities and similarities. The percentage of sequence identities, e-values and scores were noted. The proteins with the highest sequence identity percentages were selected for homology modelling. The Uniprot, EMBL-EBI and NCBI automated server that performs interactive multiple sequence alignments (MSA) were used to align and compare the amino acid sequence of human proteins like IR, GLUT and adapter proteins involved in signal transduction pathways with the ACA1_163470, ACA1_060920 and ACA1_ 176180 proteins of amoebal origin.

#### Homology modelling of ACA1_163470, ACA1_060920 and ACA1_ 176180

The SWISS-MODEL automated server^23,24^ was used to build template-based models of the *A. castellanii* proteins ACA1_163470, ACA1_060920 and ACA1_ 176180, which appeared to be the closest match of human IR and GLUTs respectively. Template-based model development was done by submitting the sequence of *A. castellanii* proteins in FASTA format as the target protein sequence. The model and template were analyzed for similarities of amino acids in the ligand-binding (glucose and ATP) pockets. The QMEAN and Ramachandran plots of the models were analyzed as well. In cases where two proteins appeared to have similar e-values and sequence identity percentages, the protein that scored better was selected for homology modelling.

#### Insulin signal transduction pathway similarities

The KEGG pathways database^25^ was searched for known human IR mediated signal transduction pathways and the adapter proteins involved in the cascade of mobilizing GLUT-4 containing vesicles, cellular proliferation, protein anabolism and events of ion-influx. *A. castellanii* genome was searched for homologs of the adapter proteins involved in IR signal transduction by BLASTp searches to establish the expression of all the downstream components that possibly mediate the effects of insulin.

### Ethical concerns and permission

This paper reports experiments that do not involve any live vertebrates, and/or higher invertebrates and human subjects. No images included in this paper require permissions from authors or journals for their reproductions. The data retrieval was done from online accessible databases and servers that are free to the public and scientists, allow publishing of the data generated by servers and have been cited in this paper.

## Results

### Insulin at a dose range of 2.98–5.97 µmol/mL causes proliferation of *Acanthamoeba* trophozoites

**Figure 1:**
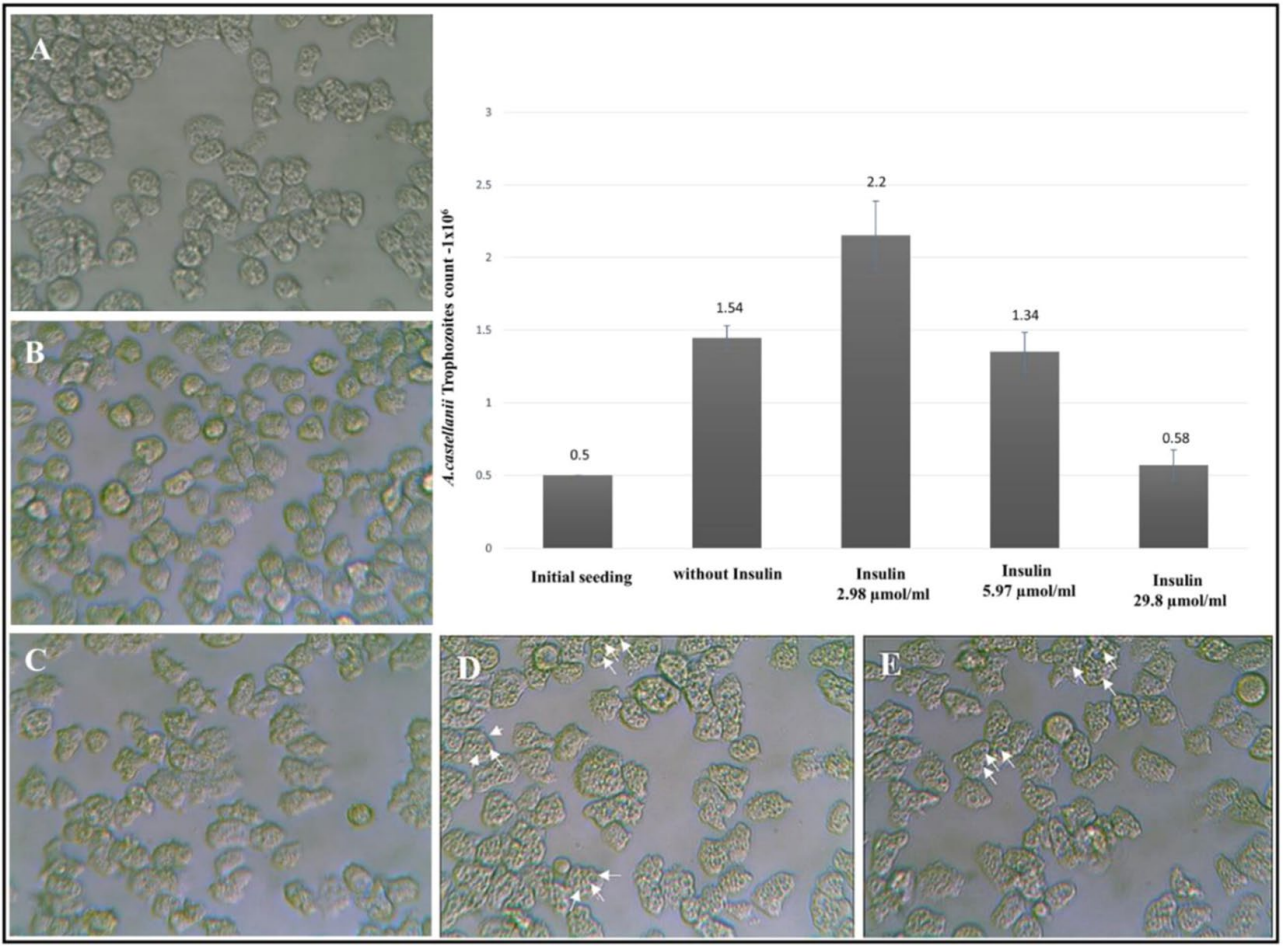
Proliferative effects of Insulin and metformin in *Acanthamoeba* trophozoites. (**A**) Trophozoites in the control, (**B,C**) are the effects of 2.98 and 5.97 µmol/mL of insulin respectively at 24 h. (**D**) Effects of metformin 100 µg/mL alone and (**E**) in combination with 5.97 µmol/mL of insulin. Note the appearance of prominent vacuoles (arrows) with exposure to metformin (**D**,**E**). The histogram shows 2.98 µmol/mL of insulin to be proliferative (B and lane-3) in contrast to effects of and 5.97 µmol/mL (near to controls) and 29.8 µmol/mL of insulin (lane-5) which shows that the counts remained close to initial seedings without proliferation. The insulin concentrations are shown in the x-axis and the response as trophozoites growth on the y-axis in the histogram. Images (40×) and the data are represented of at least three independent experiments performed in duplicates.

In growth assays, *Acanthamoeba* trophozoites (0.5 × 10^6^) incubated with human insulin for 24 h in a range of 2.98–5.97 µmol/mL (Fig. 1B,C) showed a proliferative effect. The dose of 2.98 µmol/mL exhibited more than 3-fold increase (2.25 × 10^6^) in the counts of the trophozoites as compared to controls (Fig. 1, histogram lane-3). Insulin in 5.97 µmol/mL exerted moderate proliferative effects (1.34 × 10^6^ trophozoites) as compared to that of controls (Fig. 1, lane-4 histogram). Insulin concentration of 29.8 µmol/mL did not exhibit proliferative effects as seen at 24 h (Fig. 1: lane-5 histogram)

### Insulin with metformin promotes the growth and proliferation of *Acanthamoeba* trophozoites

Insulin alone and in combination with metformin promoted growth and proliferation of *Acanthamoeba* trophozoites. *Acanthamoeba* trophozoites (0.5 × 10^6^) in controls (Fig. 1A) when incubated for 24 h with insulin 2.98 µmol/mL showed growth-promoting effects evidenced by prominent cellularity (Fig. 1B). With 5.97 µmol/mL the effects in 24 h were close to the controls. (Fig. 1C). With metformin100µg/mL alone the trophozoites showed proliferation with the appearance of prominent vacuoles within the cytosol (Fig. 1D-arrows). This effect of metformin persisted even after the addition of insulin in a dose of 5.97 µmol/mL (Fig. 1E).

### Insulin causes glucose entry in the trophozoites of *Acanthamoeba castellanii*

**Figure 2:**
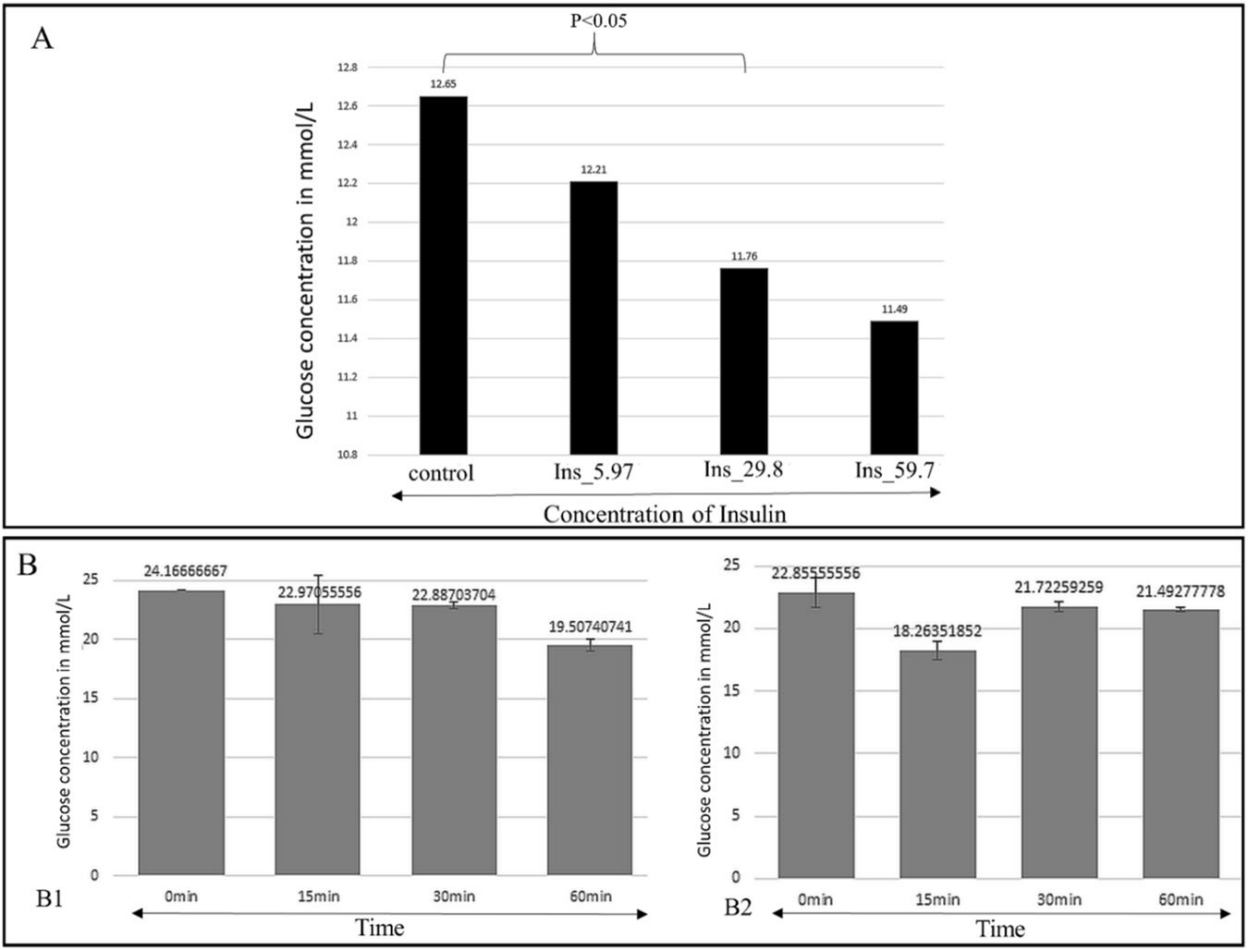
Effects of insulin on glucose entry in *Acanthamoeba* trophozoites. (**A**) 0.5 × 10^6^ *Acanthamoeba* trophozoites grown in PYG for 24 h (Lane-1) with 12.78 mmol/L of glucose at initial seeding. Effects of 5.97, 29.8 and 59.7 µmol/mL of insulin respectively (lane-2, 3 4). When estimated after 24 h, the glucose levels fell in all the well plates treated with different doses of insulin. Data are represented as the means and standard errors (P < 0.005; paired *t*-test; one-*t*ail distribution) of at least three independent experiments performed in duplicates. (**B**) 12 × 10^6^ *Acanthamoeba* trophozoites grown in PG medium treated with 35.83 µmol/mL (B1) and 47.78 µmol/mL of insulin (B2). Glucose estimations at 15 mins, 30 mins and 60 mins showed glucose levels declined in the insulin exposed wells as compared to the controls. Data are represented as of least three independent experiments performed in duplicates.

Long- and short-term effects of different doses of insulin were tested in *Acanthamoeba* trophozoites 0.5 × 10^6^ and 12 × 10^6^ respectively to provide evidence of insulin-induced glucose uptake in this unicellular eukaryote. When 0.5 × 10^6^ trophozoites were exposed to 5.97, 29.8 and 59.7 µmol/mL of insulin for 24 h the glucose level declined in all well plates. In the control wells, the glucose fell from 12.72 mmol/L to 12.65 mmol/L in 24 h (Fig. 2A lane-1). In well plates with 5.97, 29.8 and 58.7 µmol/mL of insulin the glucose concentration decreased to 12.21, 11.76 and 11.49 mmol/L respectively (Fig. 2A lane-2, 3 and 4). As the insulin in humans commences the glucose entry into the cells within about 15–30 mins after binding to IR, short-term effects were tested in 12 × 10^6^ trophozoites with insulin (Fig. 2B). Our results show that as compared to the controls (Fig. 2B1, B2–1^st^ lane) glucose levels fell in all well plates at 25, 30 and 60 mins.

### Insulin treated *Acanthamoeba* trophozoites evidence the effects of glucose entry and accumulation in the form of glycogen by increased PAS staining

**Figure 3:**
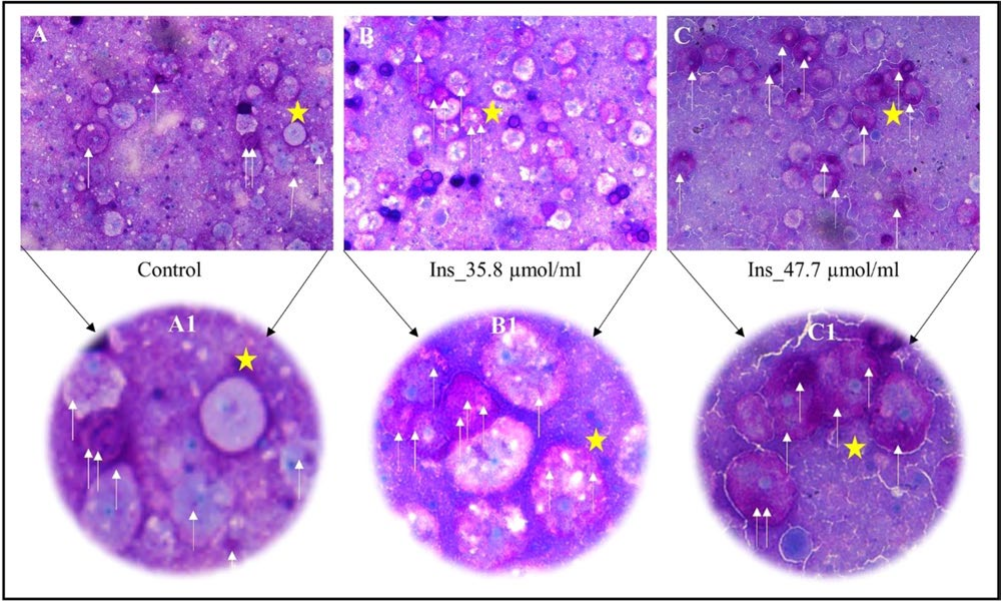
PAS staining in *Acanthamoeba* trophozoite after Insulin exposure. (**A**) *Acanthamoeba* trophozoites showing PAS staining without insulin exposure. (A1) Inset of A (yellow star) showing normal PAS stained trophozoites. (**B,C**) Enhanced PAS staining of *Acanthamoeba* trophozoites after 24 h exposure to 35.83 and 47.78 µmol/mL of insulin respectively. (B1–C1) Inset of B and C (yellow stars) showing a gradual increase in magenta-purple coloured PAS staining with increasing insulin concentrations.

To demonstrate that insulin caused the entry of glucose and accumulation of glycogen inside the trophozoites, PAS staining was done in controls (non-insulin treated trophozoites) and trophozoites exposed to insulin. The increased PAS staining in trophozoites as compared to the controls (Fig. 3) was observed when trophozoites were grown for 24 h.

### Insulin promotes K^+^ entry inside the *Acanthamoeba* trophozoites

**Figure 4:**
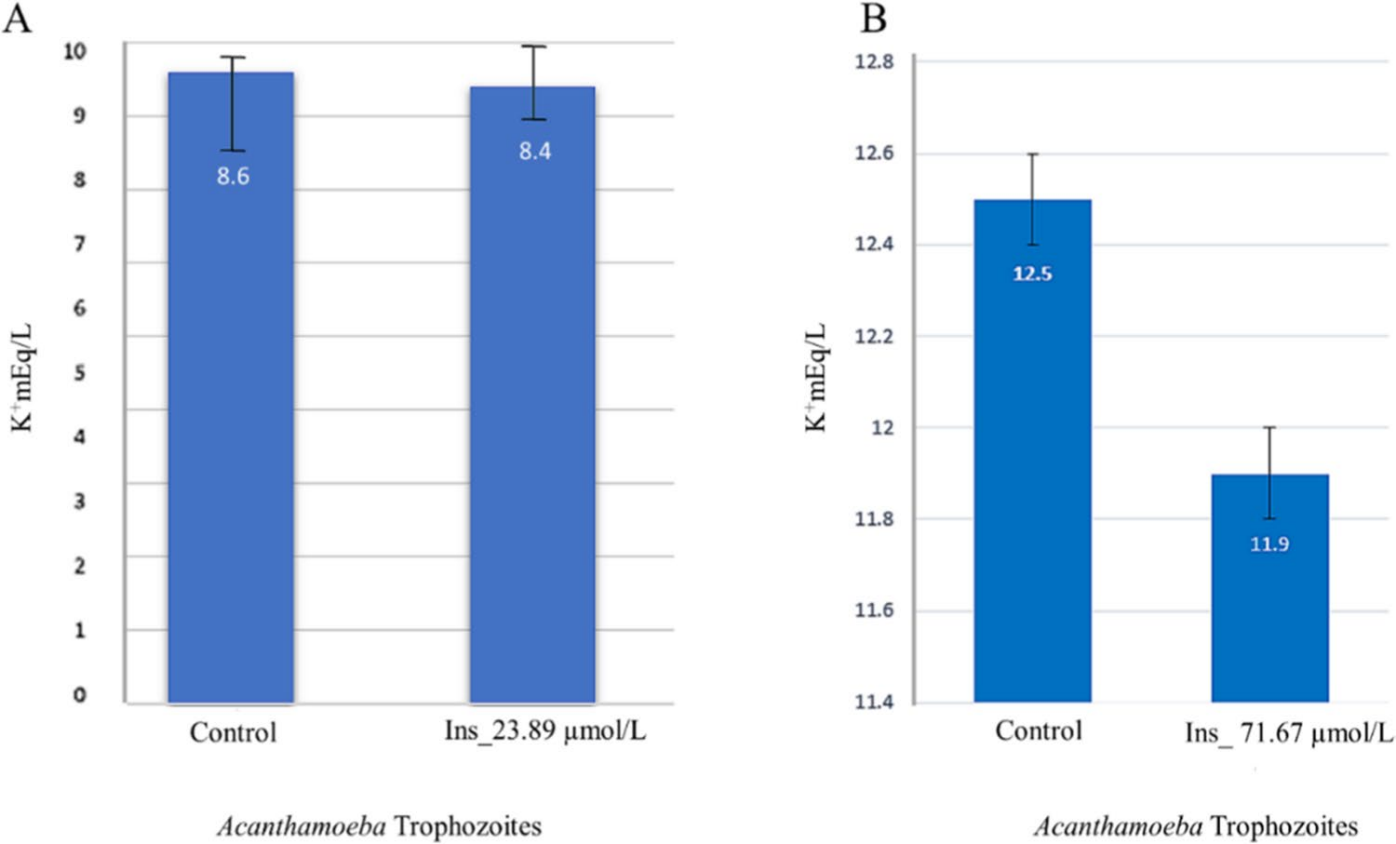
Effects of Insulin on K^+^ entry in *Acanthamoeba* trophozoites. (**A**) Insulin, 23.89 µmol/mL induced potassium entry in *Acanthamoeba* trophozoites. In 12 × 10^6^ trophozoites, (A-lane 2) showed a fall of glucose at a rate of 0.2 mEq/L per hour (**B**) A concentration of 71.67 µmol/mL (three-time higher) insulin decreased the K-levels from 12.5 to 11.9 mEq/L (0.6 mEq/L) in an hour which shows the decrease of K^**+**^ levels in the PYG medium occurs at the steady rate of 0.2 mmol/L/h on insulin exposure. Data are representative of at least three independent experiments performed in duplicates.

Insulin is known to cause K^+^ entry inside human cells. We tested insulin in *Acanthamoeba* trophozoites to observe if insulin had a similar effect, 12 × 10^**6**^ trophozoites in PYG growth medium (Fig. 4A,B first lane) were exposed to 23.89 and 71.67 µmol/mL of insulin respectively and K^+^ levels were measured after 60 mins. It was observed that K^+^ levels fell steadily at the rate of 0.2 (Fig. 4A) and 0.6 (Fig. 4B) mEq/L/h in the wells that were treated with 23.89 and 71.67 µmol/mL of insulin respectively (Fig. 4A,B lane # 2) as compared to control wells (Fig. 4A,B lane #1).

### Insulin induces Ca^2+^ entry inside the trophozoites of *Acanthamoeba castellanii*

**Figure 5:**
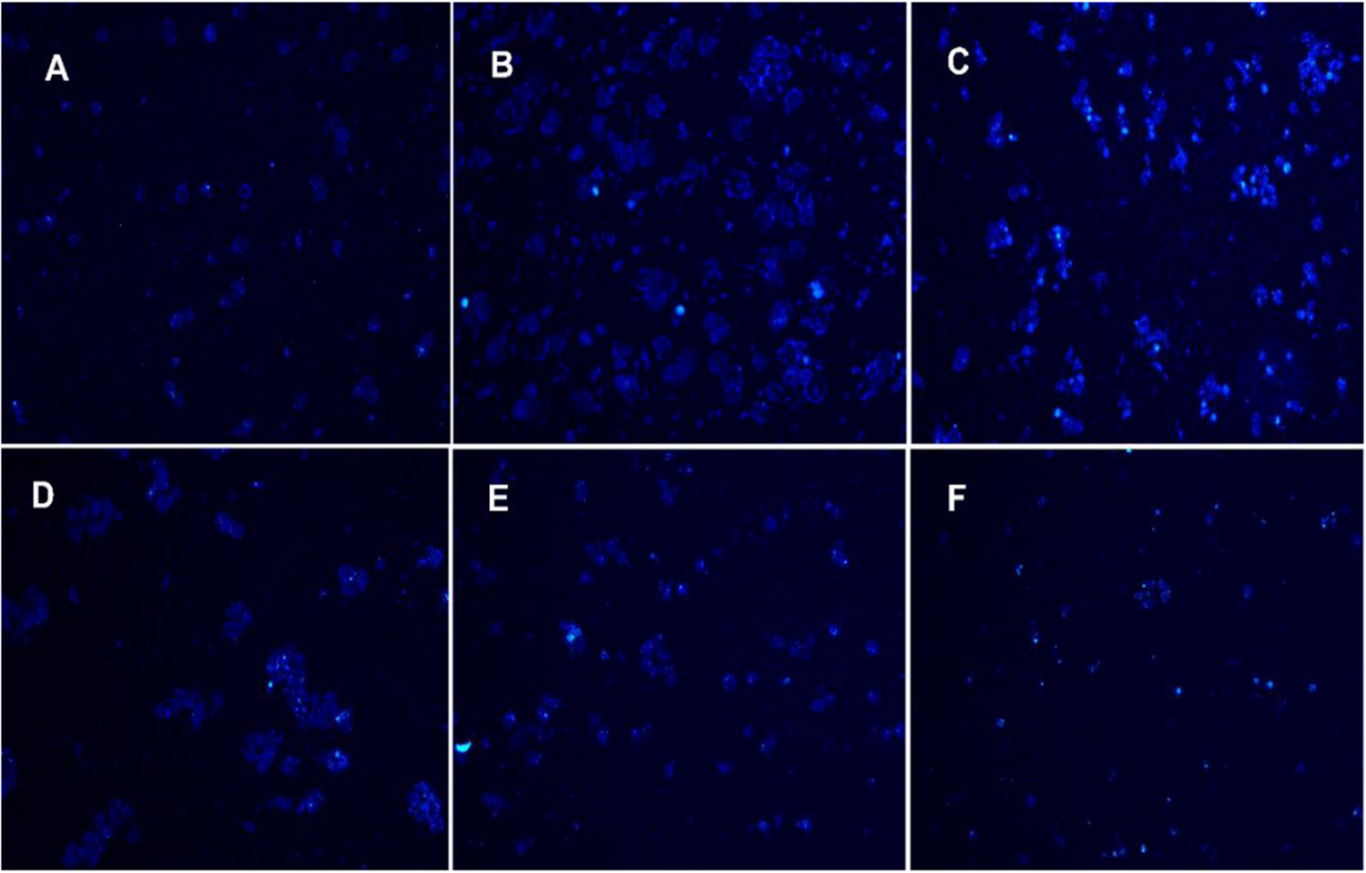
Fura2-AM staining of *Acanthamoeba* after treatment with different concentration of insulin. (**A**) Control, (**B**) Ins_0.29 µmol/mL, (**C**) Ins_2.98 µmol/mL, (**D**) Ins_5.97 µmol/mL, (**E**) Ins_29.8 µmol/mL, (**F**) Ins_59.7 µmol/mL. Data are representative of at least three independent experiments performed in duplicates.

Calcium ion entry inside human β cells of islets of the pancreas is known to stimulate insulin release, but insulin also causes calcium entry in human tissues. We show that insulin causes a dose-dependent Ca^2+^entry in the trophozoites as compared to controls (Fig. 5A), as evidenced by bright Fura2-AM staining in insulin concentrations between 0.29 µmol/mL to 5.97 µmol/mL (Fig. 5B–D). These trophozoites continued to grow when observed for next 48 h. Insulin in concentrations of 29.8–59.7 µmol/mL initially showed bright Fura-2 AM staining (Fig. 5E,F) in trophozoites, but they later failed to grow in PYG medium (data not shown).

### Insulin causes protein anabolic effects as reflected by enhanced cytoplasmic eosinophilic staining in trophozoites of *Acanthamoeba* spp

**Figure 6:**
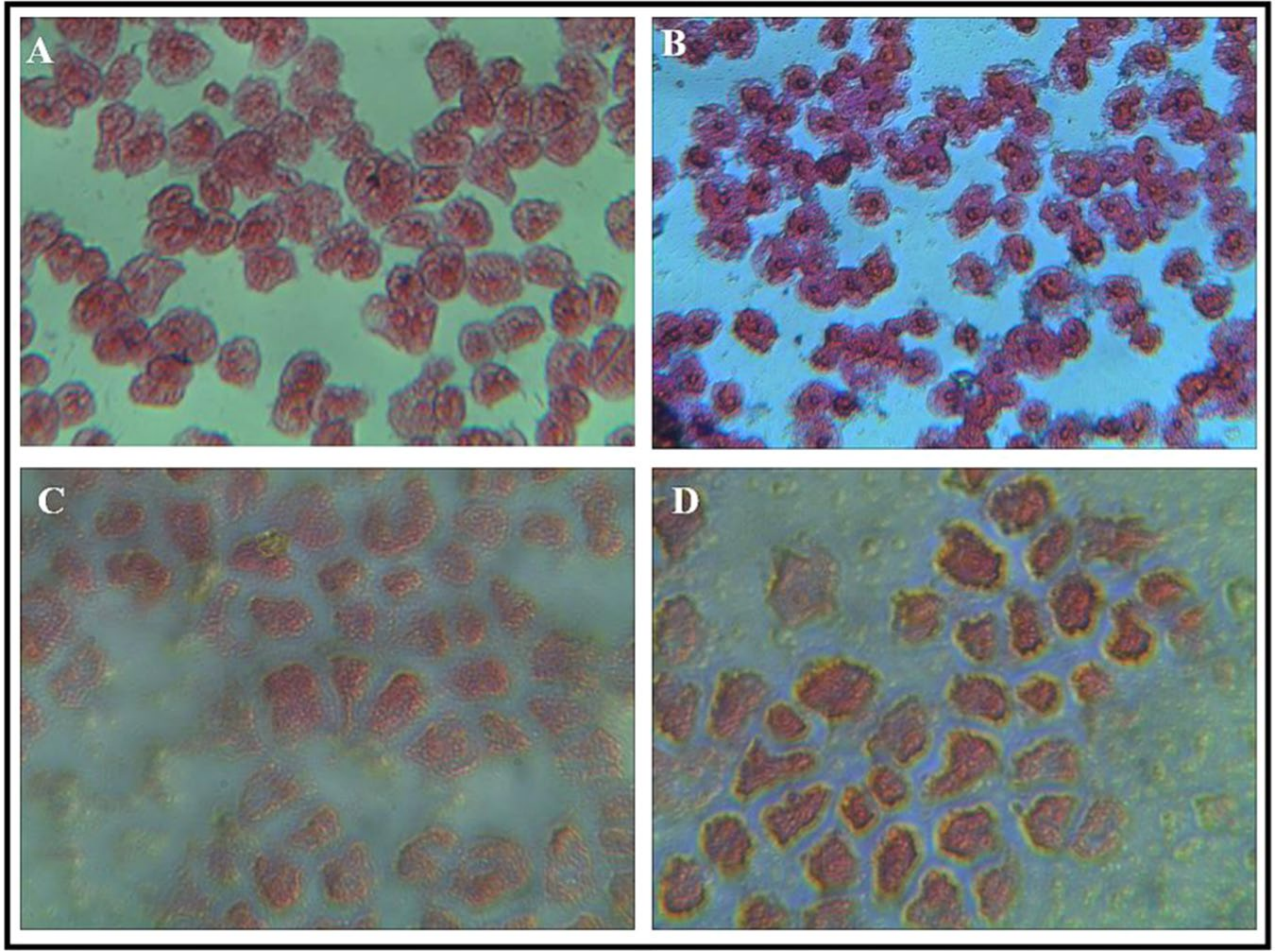
Effects of Insulin and metformin on protein anabolism in *Acanthamoeba* trophozoites. (**A**) Staining of untreated trophozoites (**B**) Trophozoites treated with 15.53 µmol/mL of insulin showed prominent cytosolic eosinophilic staining. (**C**) Metformin 100 µg/mL reduced the cytosolic eosinophilic staining as trophozoites appeared less pink. (**D**) 15.53 µmol/mL of insulin was able to restore the protein catabolism as trophozoites appeared pinker as compared to (**C**). Imaging experiments were performed in duplicate and random areas under the microscopes were selected to capture images.

Insulin via IR signalling exerts a protein anabolic effect in human cells. To validate that a similar effect occurs in *Acanthamoeba* trophozoites, different doses of insulin alone and in combination with 100 µg/mL metformin were tested. Compared to PYG control (Fig. 6A) trophozoites treated with 15.53 µmol/mL of insulin (Fig. 6B) stained more eosinophilic within their cytoplasm which is a possible reflection of a known protein anabolic effect of insulin.

Metformin is an anti-diabetic drug that is known to cause autophagy in human cells (see discussion) and was tested alone and in combination with insulin in *Acanthamoeba* trophozoites. Metformin in 100 µg/mL alone appeared to reduce the cytoplasmic eosinophilia in *Acanthamoeba* trophozoites (Fig. 6C). Interestingly, 15.53 µmol/mL of insulin was partially able to restore the eosinophilia in trophozoites treated with 100 µg/mL of metformin (Fig. 6D).

### *Acanthamoeba castellanii* protein ACA1_163470 has sequence similarities and shares functional attributes with human insulin receptor

**Figure 7:**
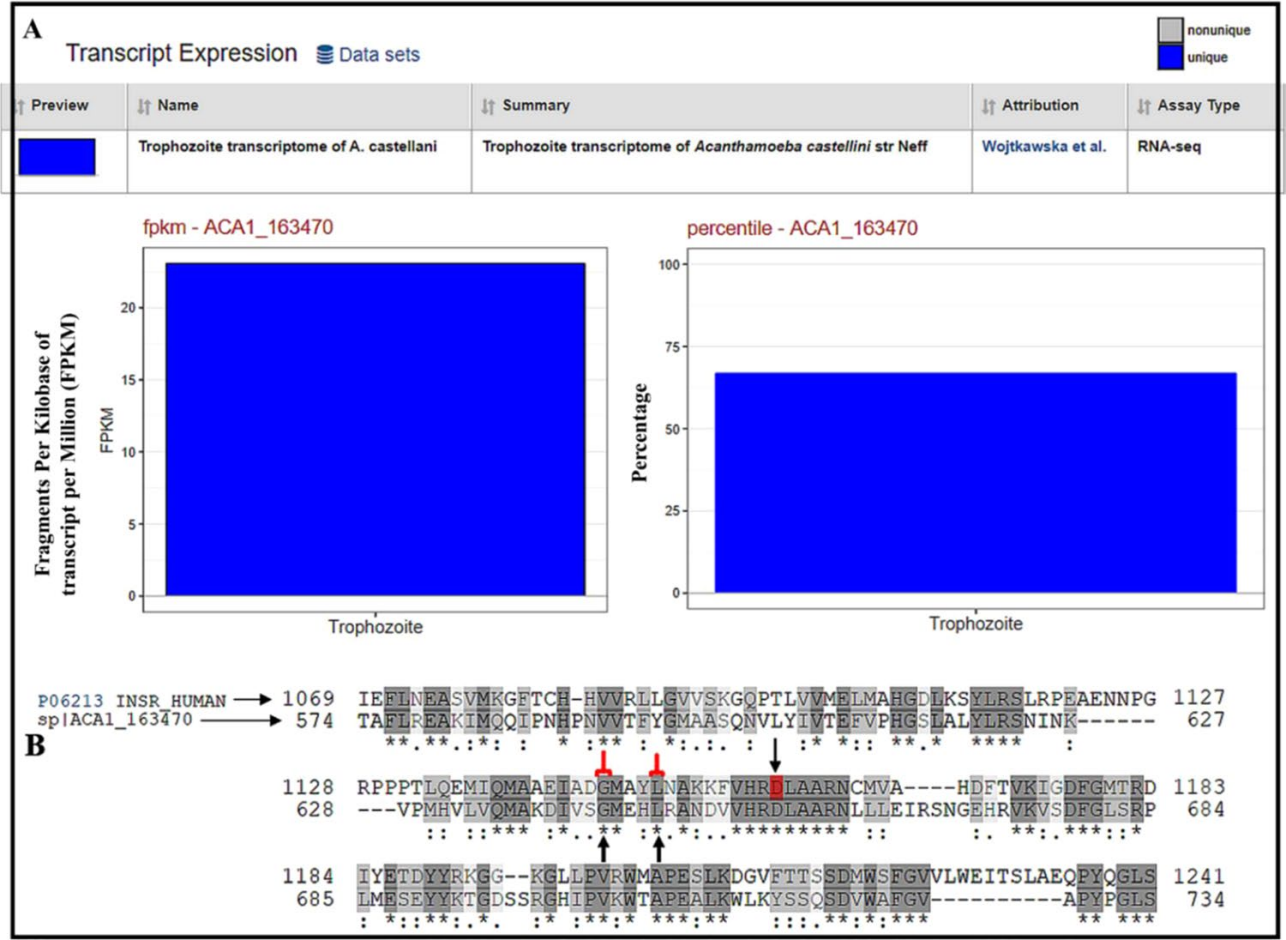
mRNA encoding and sequence similarity results for ACA1_163470. (**A**) Trophozoite transcriptome of *A. castellani* showing mRNA encoding ACA1_163470 retrieved from AmoebaDB.org is shown in fragments per kilobase of exon model per million mapped reads (FPKM) (A- left) and percentage (A- right). (**B**) Uniprot automated server was used for sequence similarity search and alignments. The aligned sequences of human IR (top-row) and ACA1_163470 (bottom-row) showed identical amino acids at 1146^th^ and 1150^th^ position (vertical arrows and red brackets) which are known to be essential for the kinase activity of human IR. The inverted arrow in the second row at 1159^th^ position (red-alphabet) is a designated active site in INSR_HUMAN (human IR), active site represents amino acid (s) directly involved in the activity of an enzyme which is identical to the amino acid residue detected in ACA1_163470 (bottom-row). [Screenshots of transcriptomics and sequence alignments were retrieved from AmoebaDB.org server^14^ and Uniprot^15^ respectively].

The sequence of human IR (accession number- P06213) was used to search for a homolog protein in *Acanthamoeba* databases that fetched *Acanthamoeba* ACA1_163470 (a protein kinase domain-containing protein) as the closest match in BLASTp search results. The transcriptomics of *Acanthamoeba* trophozoites shows the expression level mRNA encoding this protein in the trophozoites (Fig. 7A). Sequence alignment of human IR with ACA1_163470 showed identical amino acids at positions 1146–1150 (Fig. 7B two vertical arrows) which are known catalytic (amino acid number 1146, 1150, 1151) domain needed to promote the kinase activity of the IR. Also, the amino acid in human-IR that is known to be directly involved in an enzyme-like activity of this protein was identical to the amino acid in ACA1_163470 (Fig. 7B, inverted arrow in the 2^nd^ row).

### Homology modelling shows ACA1_163470 in *Acanthamoeba castellanii* to be a homolog of human IR

**Figure 8:**
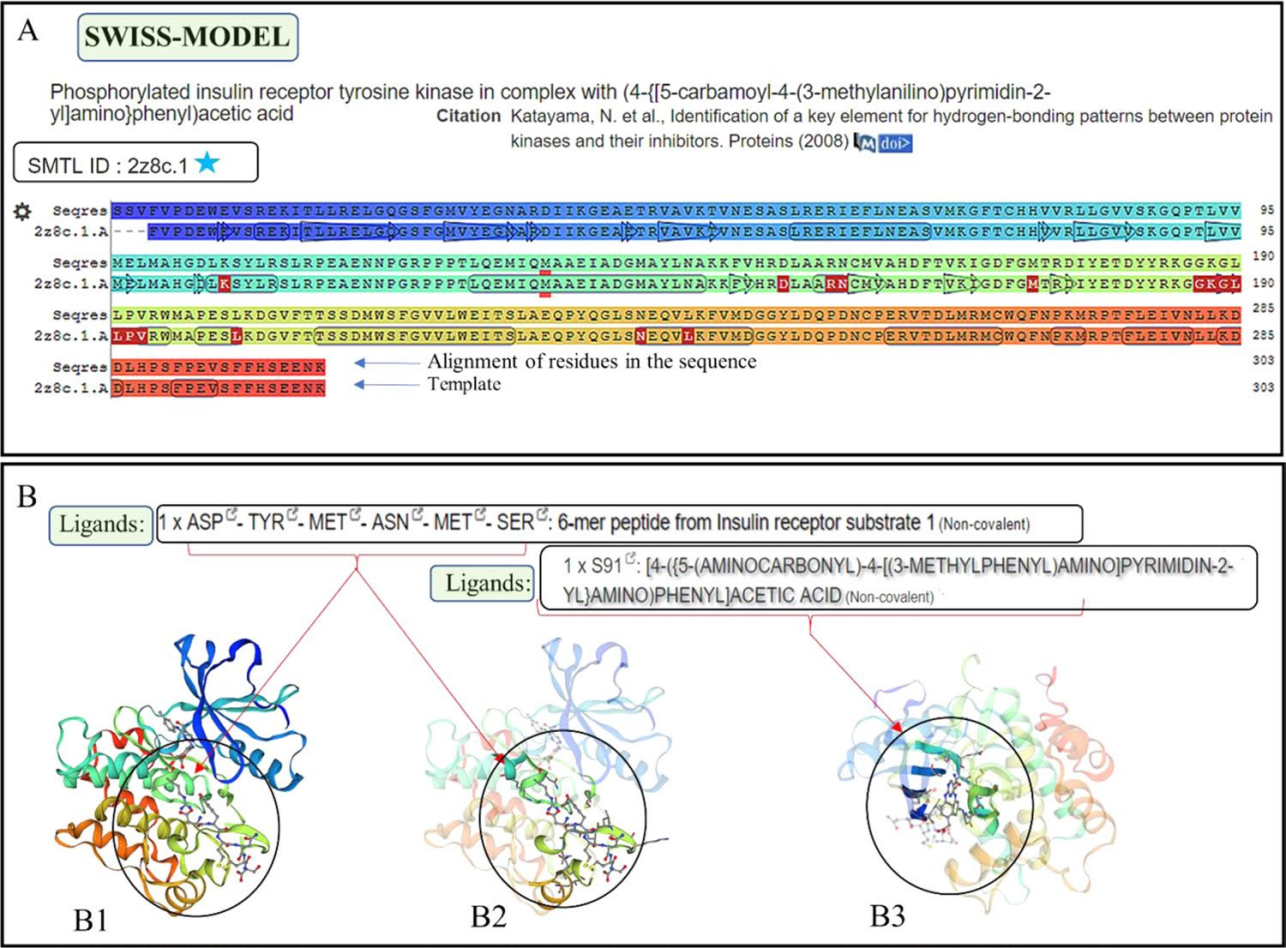
Homology modelling results of ACA1_163470. (**A**) The FASTA sequence of *Acanthamoeba* protein ACA1_163470 was submitted to SWISS-MODEL automated server for developing a template-based model. Coloured rows show the similarities between template generated for ACA1_163470 and model developed (**B**) The SWISS-MODEL database used the template (2z8c.1)-to build a model (rainbow structures) that have identical ligand-binding residues (circles). [Screenshots of template-model retrieved from SWISS-MODEL server^23,24^].

SWISS-MODEL automated server^23,24^ developed a template-based model for *Acanthamoeba* ACA1_163470 which has similarities with human IR. The template (PDB ID - 2z8c.1.A) was used in the SWISS-MODEL server to build a model for amoebal protein (Fig. 8A) which was recognized as phosphorylated IR bound to ligands (Fig. 8B). The model developed for ACA1_163470 showed ligand-binding pockets (Fig. 8 B1–B3) with identical amino acids residues shared between the template for ACA1_163470 and model human IR (Fig. 8A - Seqres and 2z8c.1 rows).

### Homology Modelling of *Acanthamoeba* ACA1_336150 developed a template-based model of human IGF1-R

**Figure 9:**
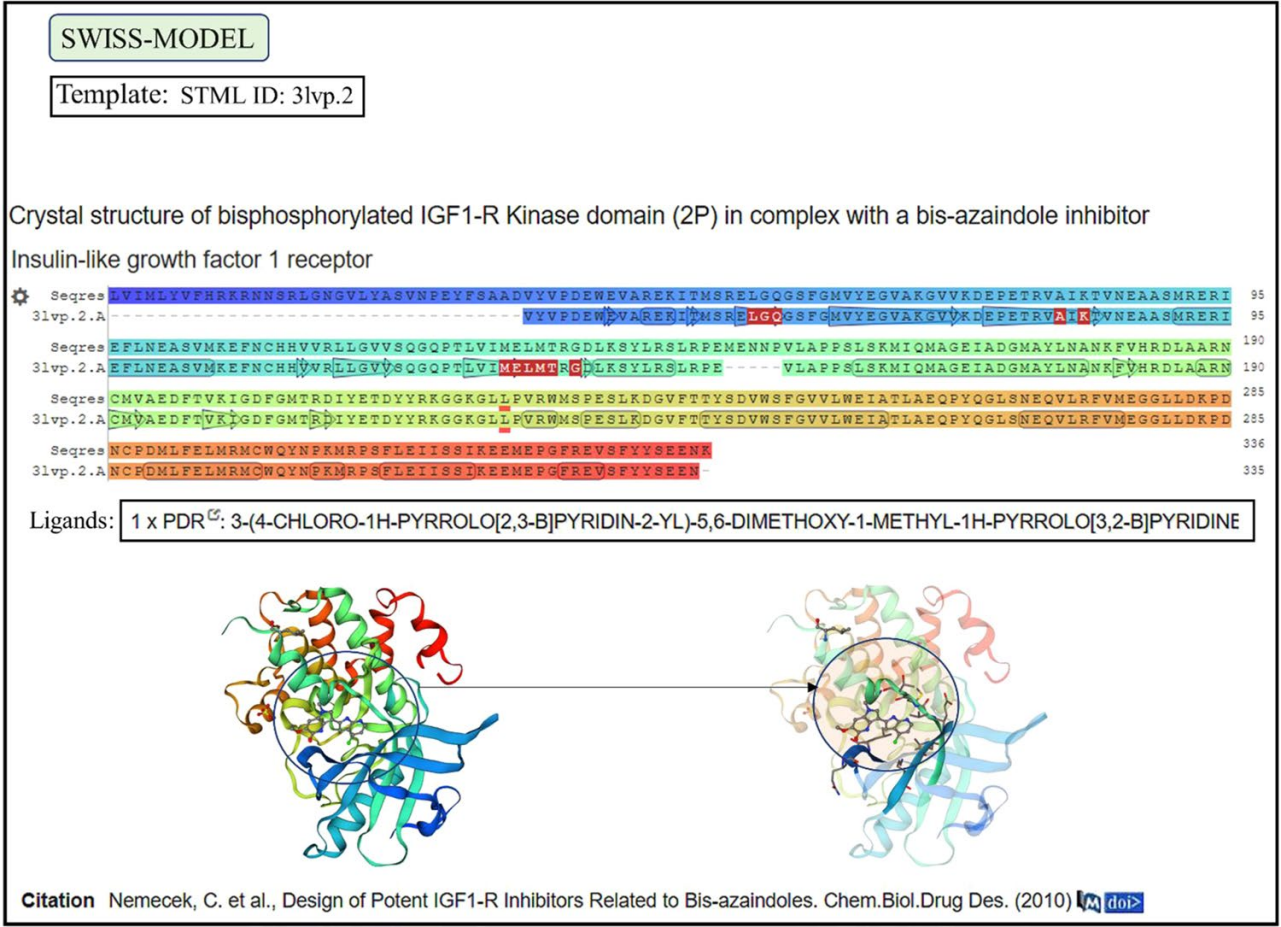
Homology Modelling of ACA1_336150. Homology modelling of ACA1_336150 built a template-based model of human IGF1-R with a template having a PDB ID 3lvp.2. Aligned with the model the sequences show identical ligand (PDR)-binding residues (coloured rows -Seqres and 3lvp.2). The pocket for ligand engagement is revealed in the model IGF1-R as shown in the solid and transparent rainbows (circle with arrow). [Screenshots of template-model from retrieved from SWISS-MODEL server^23,24^].

On submitting the FASTA sequence of *Acanthamoeba* protein ACA1_336150 to the SWISS-MODEL automated server for homology modelling, a template-based model was developed. The template used to build a model has a PDB ID 3lvp.2 (Fig. 9) which was recognized as the IGF1-R kinase domain bound to ligand (Fig. 9 models with the circled area). The model of human IGF1-R showed ligand-binding pockets (Fig. 9- rainbow models at the bottom) with identical amino acids residues shared between the template and model that engage the ligands (Fig. 9- Seqres and 3lvp.2. coloured rows). Transcriptomics data retrieved for mRNA encoding ACA1_336150 showed to be above 50% (Supplementary File-Fig. 1).

### Protein ACA1_060920 and ACA1_ 176180 in *Acanthamoeba castellanii* has sequence similarity and attributes like human GLUT channels

**Figure 10:**
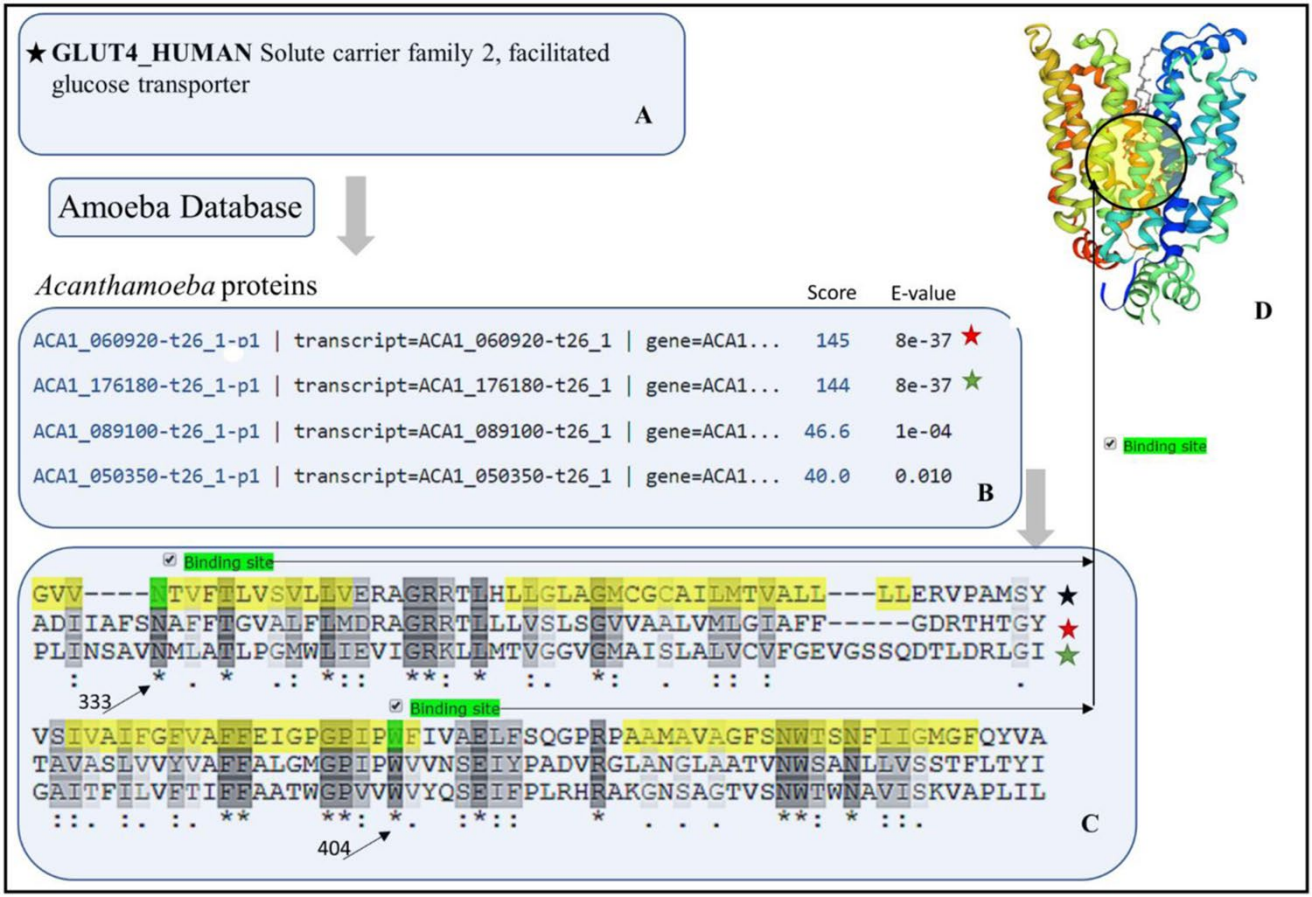
Sequence similarities and ligand binding attributes of human GLUT and *Acanthamoeba* proteins. (**A**) Sequence similarities between human GLUT-4 (black star) and (**B**) *Acanthamoeba* homolog proteins ACA1_060920 and ACA1_176180 (red and green stars respectively). (**C**) Alignment of the sequences shows similar glucose (oblique arrow top row) and ATP binding sites (oblique arrow, bottom row). Also shown are the location of transmembrane regions (yellow) and ligand binding sites (green) in sequence, with 333^rd^ for glucose and 404^th^ position for ATP respectively. (**D**) Structural model of human GLUT1 (3D model at top-right) is shown with the ligand-binding pocket. [Screenshots of BLASTp, MSA and structural model retrieved from Amoeba.DB.org^14^, Uniport^15^ and SWISS-MODEL server^23,24^].

A BLASTp search for finding homologs of human-like GLUT proteins (Fig. 10A) in *Acanthamoeba* genome deposited at AmoebaDB.org database, retrieved four proteins as a match for human GLUTs (Fig. 10B). The *Acanthamoeba* proteins ACA1_060920 and ACA1_176180, when aligned with human GLUT-4, exhibited the presence of identical amino acids within the ligand-binding active region in their sequence (Fig. 10C). The complete amino acid sequence of protein ACA1_060920, when aligned with human GLUT-4 protein, showed the ligand (glucose) and ATP binding amino acid residues in human GLUT-4 sequence were identical with the residues in sequence of the ACA1_060920 located within the transmembrane region (Fig. 10C, yellow highlighted regions-333^rd^ and 404 amino acids) of the sequences compared (Fig. 10C). Transcriptomics of ACA1_060920 and ACA1_176180 (Supplementary File-1 A,B) showed in fragments per kilobase of exon mode per million (FPKM) and percentages appeared to be around 22% and 30% respectively. Although the crystal structure of GLUT-4 is not deposited in the PDB database, the sequence of GLUT-4 and other GLUTs in human are known. We aligned the human GLUTs with ACA1_060920 to show that the Asparagine (N) known to bind ligand (glucose) and ATP are identical (Fig. 10C, 333^rd^ and 404^th^ position respectively) between all known human GLUTs and ACA1_060920 (Supplementary File: Fig. 3). The GULT-1 model is shown (Fig. 10D) with the pocket that is known to bind the ligands.

### Insulin receptor downstream signalling pathway has similarities between human and *Acanthamoeba* spp

Comparative IR signalling pathway similarity search between humans and *Acanthamoeba* spp. showed near-identical adapter proteins that possibly elucidate the results obtained with insulin as reported in this study. Adapter proteins involved in mediating glucose entry, K^+^-influx, Ca^**2+**^ movement inside the cells, protein anabolic effects, proliferative stimuli and growth promotion were found to be expressed in *Acanthamoeba castellanii*.

**Figure 11:**
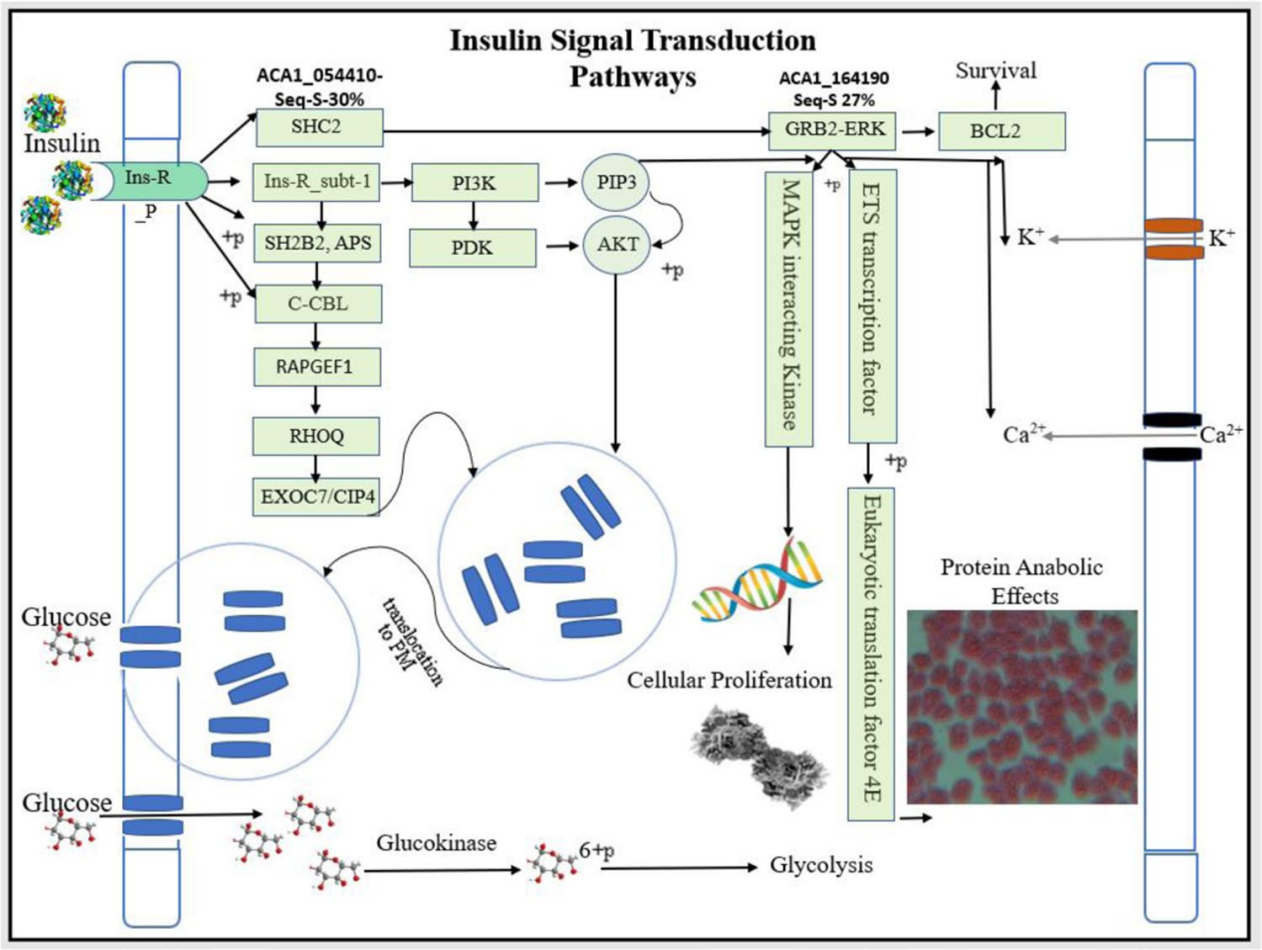
Insulin receptor adapter proteins similarities between human and *Acanthamoeba* spp. The adapter proteins (green boxes) in human cells are known to mediate IR signalling cascade are already known. The search for homologs of the human IR signalling adapter proteins in AmoebaDB.org fetched ACA1_ proteins (Table 1; 2^nd^ column) that possibly mediate the mobilization of GLUT-4 like protein from the vacuole to the cell membrane, cause K^**+**^ /Ca^2+^ influx, mediate the spp. protein anabolic effects and proliferation in *Acanthamoeba* spp.

A search for homologs of human IR signalling proteins (Fig. 11- green-boxes) in the database^25^ showed adapter proteins that had a very high sequences identity percentage with similar proteins in *Acanthamoeba* spp. (Table 1). The scores, sequences identity percentages and e-values obtained on BLASTp results show the amoebal proteins to the homologs of the human adapter proteins involved in IR signalling pathway.

**Table 1:** Human proteins involved in IR signalling and their homologs in *Acanthamoeba* spp.

Human IR Signalling Adapter Proteins	Homologs in *Acanthamoeba* spp	Percentage of Sequence Identities	E-value/QMEAN
Ins_R_sub-1- The ‘pleckstrin homology’ (PH) domain	**ACA1_281600-** PH domain containing protein	32.11%	QMEAN −3.36
APS- SH2B adaptor protein 2	**ACA1_336880** -SH3 domain-containing protein	33.88%	8e-33
CBL- E3 ubiquitin-protein ligase	**ACA1_362540-** E3 ubiquitin-protein ligase AMFR	40%	1e-83
RAPGEF1- Rap guanine nucleotide exchange factor	ACA1_112150- RasGEF domain containing protein	52%	1e-45
RHOQ- Rho-related GTP-binding protein	**ACA1_064820 –** Rho family, small GTP binding protein	77%	4e-83
CIP4 - SH3_1- thyroid hormone receptor interactor 10	**ACA1_215920**- SH3 domain containing protein	43%	2e-21
PI3K- PI3-kinase	**ACA1_155440**- phosphoinositide 3kinase family	39%	5e-93
AKT3 protein, partial [Homo sapiens]	**ACA1_157980** protein kinase domain containing protein	50%	2e-108

## Discussion

Evidence of insulin-like peptides, IR and IGF-like receptors have been researched in prokaryotes and early life-forms^1,26^. Despite insulin being one of the most extensively studied hormones in humans^27^, the origin and expression of IR, IGF1-R and GLUT-like proteins in primitive unicellular eukaryotes has not been investigated in detail. We selected *Acanthamoeba castellanii* T4 genotype, one of the first single-celled eukaryotes that populated the planet about 2 billion years ago^28^, to study glucose homeostasis and possible expression of IR, IGF1-R and GLUT-like receptor proteins. Insulin causes proliferation and growth of somatic cells^29,30^ in humans. Similarly, insulin in doses of 2.98–5.97 μmol/mL promoted proliferation and growth of *Acanthamoeba* trophozoites as compared to controls (Fig. 1), while increasing doses caused the counts to remain close to the initial seedings. Metformin enhances the binding of insulin to human cells^31^. In order to observe any synergistic effects with insulin, Metformin was tested in combination with insulin in *A.castellanii*. Additionally, metformin acts on human cells to induce cytosolic vacuoles due to autophagy^31,32^. Comparably, large trophozoites with more cytosolic vacuoles (Fig. 1D) were observed in trophozoites exposed to this drug vs controls. Given with insulin, the metformin-treated cells showed persistence of cytosolic vacuoles (Fig. 1E) with a slightly increased cellular count. Insulin promotes the entry of glucose inside human cells^33–35^. Like its effects on human tissues, insulin promoted glucose entry inside the treated cells as evidenced by the declining levels of glucose in the growth medium peptone and glucose (PG medium) over a period of 24 h as well as at 15 mins, 30 mins and 60 mins after exposure to insulin (Fig. 2). Long-term (Fig. 2A) and short-term (Fig. 2B) effects of insulin were observed, with both showing a decline in glucose concentrations. Importantly, being *in vitro* tests of insulin on cell lines, concentrations of insulin and configuration of the medium were optimized. Furthermore, “non-toxic” mediums devoid of 2-deoxyglucose were utilized, as it is known to inhibit glucose influx inside the cells. Assays were done in PG (peptone and glucose) with pH 7.4 that provided an ideal medium for insulin to be tested in *Acanthamoeba* trophozoites. As PBS without nutrients and glucose triggers the process of encystation (a dormant state) in *Acanthamoeba* trophozoites^36^, we used PYG or PG medium in the 24 h assays. PAS staining was performed in trophozoite controls and those treated with insulin in order to elucidate the entry of the glucose inside the cells and its subsequent storage in form of polysaccharides, as this process is induced by insulin. Enhanced PAS staining was observed in the insulin-treated trophozoites as compared to controls (Fig. 3).

High K^+^ levels were noted in PYG with trophozoites in contrast to K^+^ levels in the human extracellular fluid(ECF). The cationic compounds in a medium containing yeast (a component of PYG, see methods) could have contributed to elevated K^+^ levels in our experiments, as this was noted in a previous study^37^. In humans, insulin is used intravenously with dextrose water to treat hyperkalemia, as it causes K^+^ entry inside the cells^38–40^. This same effect was tested and noted in *Acanthamoeba* trophozoites (Fig. 4). We observed consistency in the declining rate of K^+^ levels after insulin exposure, in about 12 million *Acanthamoeba* trophozoites grown in PYG when exposed to increasing levels of insulin (Fig. 4A,B).

Insulin has also been reported to increase cytosolic calcium^41–43^ in human and mammalian cells. *Acanthamoeba* trophozoites were studied for Ca^2+^ entry inside the cell after insulin exposure (Fig. 5). Differential doses of insulin promote increased intracellular free calcium in *Acanthamoeba*, as evidenced by Fura-2AM staining before (Fig. 5A) and after its exposure to insulin (Fig. 5B-E). Protein anabolic effects of normal insulin levels also contribute to cellular proliferation and growth of the human body^44,45^. Cells become more eosinophilic (acidophilic) through insulin-mediated protein anabolism, which has been observed after H&E staining^46^. Again, mimicking human cells, insulin treated *Acanthamoeba* trophozoites, showed enhanced cytosolic eosinophilia compared to the controls (Fig. 6A,B). Metformin treated trophozoites reduced the cytosolic eosinophilic staining (Fig. 6C), which was reverted to some extent by 15.53 μmol/mL of insulin (Fig. 6D). The observed effect of metformin confirms that the appearance of vacuoles is the autophagic digestion of cytosolic protein, which is a part of the process of autophagy^32^ and also appears to cause the reduced eosinophilic staining in *Acanthamoeba* trophozoites (Fig. 1D). After observing the target effects of insulin in *Acanthamoeba* spp., continued searching for an IR, IGF1-R and GLUT-like human proteins in this unicellular eukaryote resulted from natural curiosity.

Using bioinformatics computational tools, genomics/transcriptomics and homology modelling we report possible IR, IGF1-R and GLUT homologs of human proteins in *Acanthamoeba* spp. The protein ACA1_163470 is a protein kinase domain-containing protein, which has about 65% expression of mRNA encoding this protein in *Acanthamoeba* spp. On sequence alignment with human IR, this protein showed identical active sites (Fig. 7B) and ligand binding sites (Fig. 7B vertical black arrows). The protein ACA1_163470 has ankyrin repeat domains. On multiple sequence alignment, the human IR, human ankyrin repeat protein (ANK2_HUMAN Ankyrin-2) and ACA1_163470 showed regions of similarities in the transmembrane regions, binding and active sites between these proteins (Supplementary File-Fig. 4). Sequence similarity of protein ACA1_163470 showed a limited sequence similarity between ACA1_163470 and human IR (Fig. 7B grey rows). However, on homology modeling, a template (PDB ID: 2z8c.1)-based model of phosphorylated IR tyrosine kinase (Fig. 8A,B) bound to ligand was developed for this amoebal protein, and showed identical ligand binding amino acid residues (Fig. 8A rows with highlighted alphabets) between the template and the model (Fig. 8 B1–B3).

Protein ACA1_336150 was the closest match for human IGF1-R, with 50% expression of the mRNA encoding it in *A. castellanii* trophozoites (Supplementary File-Fig. 1). The template built by SWISS-MODEL automated server^23,24^ on homology modelling for ACA1_336150 has a PDB ID 3lvp. 2 (Fig. 9), which was recognized as IGF1-R kinase domain bound to ligands (Fig. 9 bottom row showing models). Aligned with the model, the template sequences show identical ligand-binding amino acid residues (Fig. 9 rows -Seqres-3lvp.2). Identical amino acid residues can also be seen between the template and the model in the pocket for binding ligand (Fig. 9, circles with the arrow on models).

Attention was then focused on finding a human-like GLUT-4 protein in *Acanthamoeba* databases, as this protein is known to get mobilized in human cells after insulin binding to IR^47^ BLASTp results showed ACA1_060920 and ACA1_176180 as homologs of human GLUT-4 (Fig. 10). Sequence alignment of human GLUT 4 and protein ACA1_060920 showed identical ligand binding amino acid residues. Multiple sequence alignment of the known human GLUTs and amoebal ACA1_060920 showed identical glucose and ATP binding sites in between these proteins (Supplementary File. Figure 3). Protein ACA1_176180 also appeared as a GLUT homolog (Fig. 10B,C -green stars), with a high sequence identity with human GLUTs. Subtypes 1–4 of human GLUT molecules are expressed at cellular levels and share sequence identities among themselves. Amoebal ACA1_060920 (red star) and ACA1_176180 (green star) displayed similar attributes (Fig. 10C).

Identifying downstream signaling adaptor proteins that ignite the cascade of insulin binding to IR has pivotal significance in proving the existence of functional IR and classifying the role of insulin in glucose homeostasis in *Acanthamoeba* spp. We searched and discovered homologs of known human IR related proteins involved in signaling cascade^13,25^ (Fig. 10 green-boxed proteins) in *Acanthamoeba* databases^14,15^. We showed the existence of adaptor proteins that are near-identical to humans, and carry forward the IR and insulin binding signaling cascade in *Acanthamoeba* spp. When compared, the sequence identity percentages of the adapter proteins were observed to be between 32%-77%, with high scores on BLASTp results and on MSA (Fig. 11 and Table 1).

An insulin-degrading enzyme present in humans, was also found to have a homolog ACA1_074110 in *A. castellanii*, these two proteins when compared had a high percentage of sequence identity and structural homology (Supplementary.File-Fig-5A and B). Our experiments, coupled with bioinformatics computational tools and homology modelling, have provided the first evidence of IR, IGF1-R and human GLUT like proteins in *Acanthamoeba* spp. This data provides intriguing clues towards the origins of the glucose homeostatic system, potentially dating back billions of years in a single-celled eukaryote, *Acanthamoeba* spp.

## Conclusion and Future Directions

This is the first report to describe and clarify glucose homeostasis regulating mechanisms in a 2 billion year old eukaryote, *Acanthamoeba castellanii*. The presence of various subtypes of human GLUT homologs like ACA1_060920 and ACA1_176180 in *Acanthamoeba* spp. explain glucose uptake mechanisms and survival of this protist in mediums like PG and PYG. Further research is needed to explore the IR and IGF1-R like proteins reported here, including their ligands in *Acanthamoeba* that regulate glucose influx.

## Supplementary information

Supplementary Information.
