# Supplementary material for: First Reports of Effects of Insulin, Human-like Insulin Receptors and Adapter Proteins in Acanthamoeba castellanii
Source: Sci Rep. 2020 Jul 16;10:11759. doi: 10.1038/s41598-020-63435-4 (PMC7366918; doi:10.1038/s41598-020-63435-4)
Supplement: Supplementary file 1 — Supplementary Information. [file 41598_2020_63435_MOESM1_ESM.pdf]

# First Reports of Effects of Insulin, Human-like Insulin Receptors and Adapter Proteins in *Acanthamoeba castellanii*

Abdul Mannan Baig[1*], Areeba K[1]

[1]Department of Biological and Biomedical Sciences, Aga Khan University, Pakistan;

Short title**: Evidence of Insulin Receptor and IGF1 Receptors in *A. castellanii***

*Corresponding address: Department of Biological and Biomedical Sciences, Aga Khan University. Tel: +92-(0)333-2644-246.

E-mail: abdul.mannan@aku.edu

## Supplementary File. Fig-1

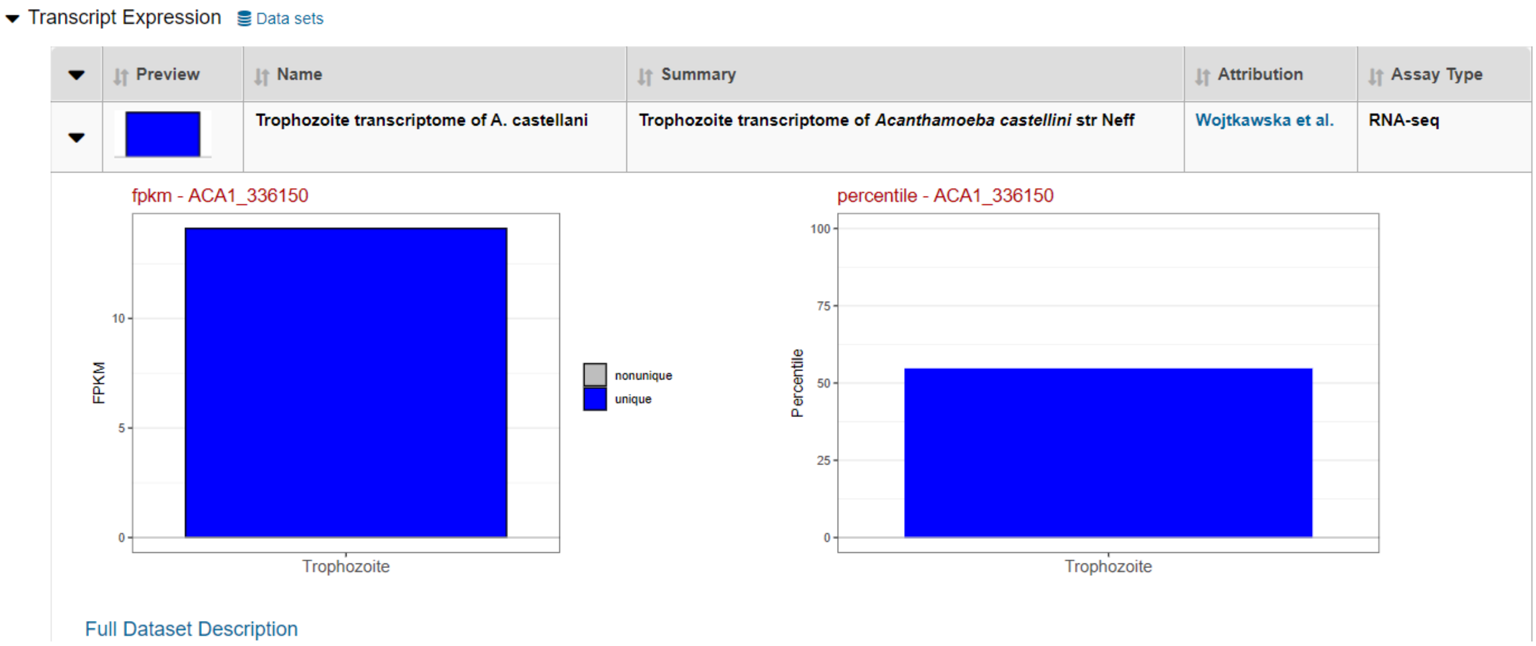


**Figure 2.** Transcriptomic of *A. castellanii* trophozoite showing expression of mRNA encoding ACA1_336150 in FPKM and percentage. **[Screenshots of transcriptomics were retrieved from AmoebaDB.org server (reference #15)]**

# Supplementary File. Fig 2

A

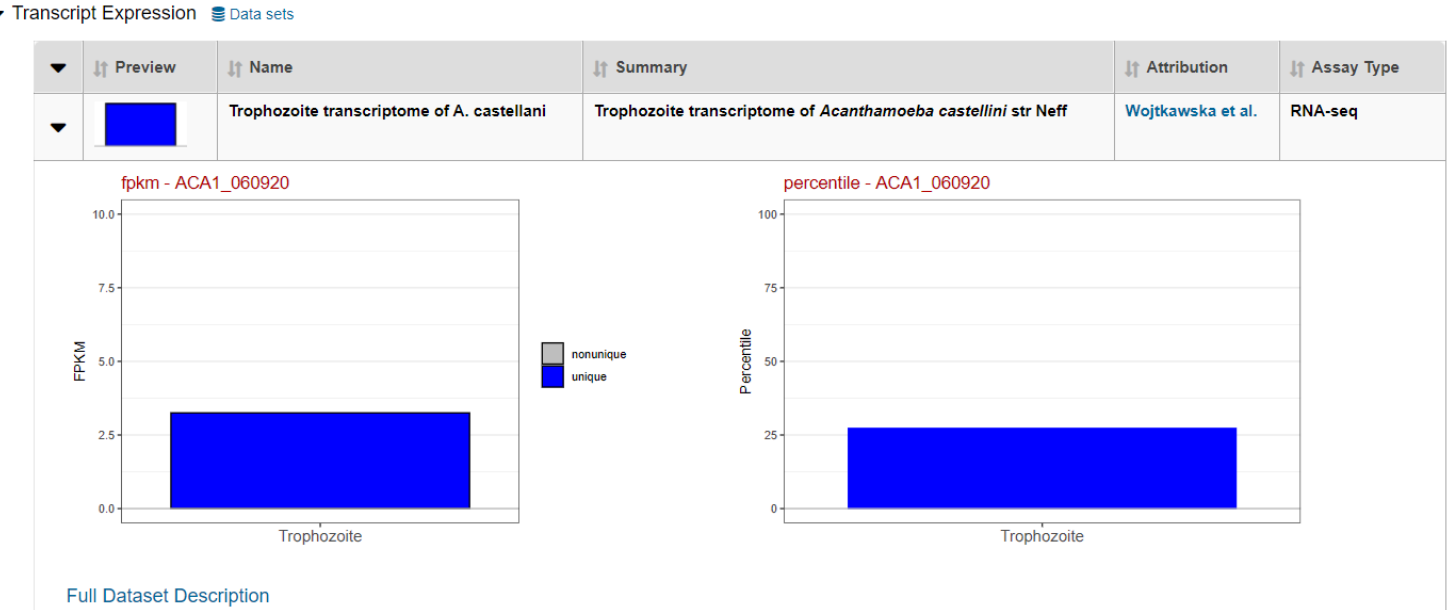


B

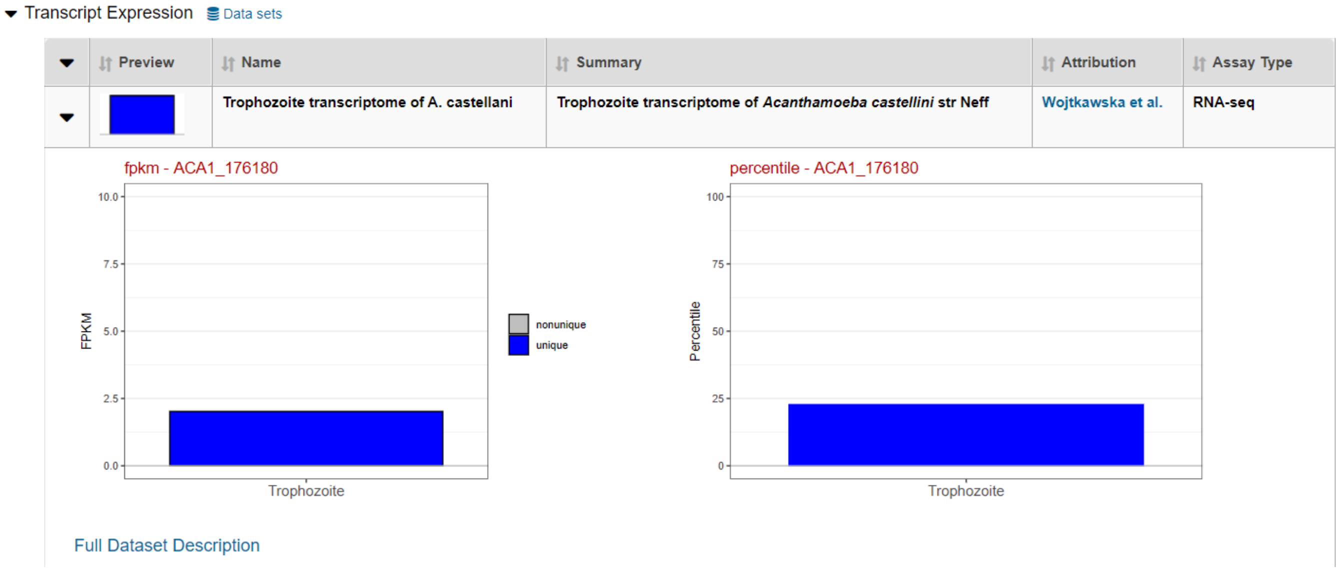


**Figure 2.** Transcriptomic of *A. castellanii* trophozoite showing expression of mRNA encoding ACA1_060920 and ACA1_176180 in FPKM and percentage. **[Screenshots of transcriptomics were retrieved from AmoebaDB.org server (reference #15)]**

**Supplementary File. Fig 3**

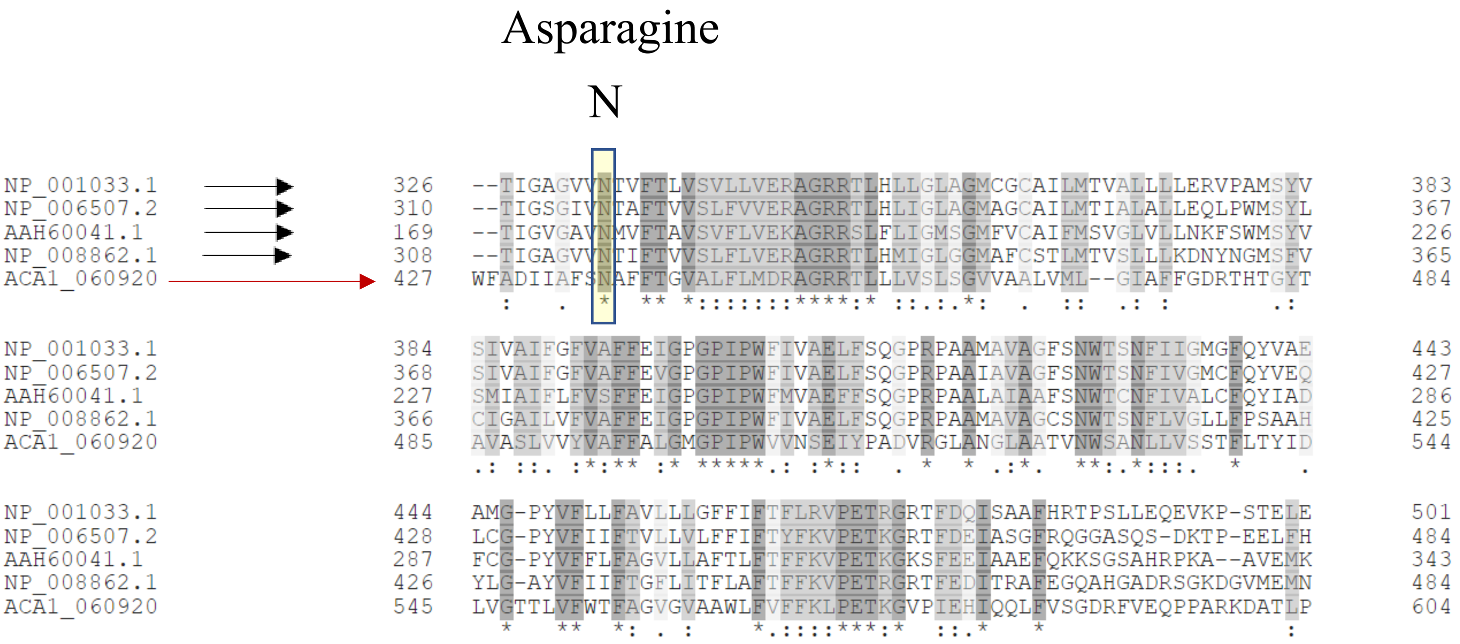


**Figure 3.** Multiple sequence alignment of human GLUT 4, 1, 2 and 3 (black-arrows from top to bottom) respectively with *Acanthamoeba* ACA1_060920 (red-arrow). Note the Glu (ligand) binding asparagine (N) is identical between human GLUTs and the amoebal protein ACA1_060920 inferred to be GLUT-4 homolog in *Acanthamoeba* spp. **[Screenshots of BLASTp and MSA retrieved from Uniport (reference #16)]**

## Supplementary –File-Fig 4

- ☑ Transmembrane
- ☑ Binding site
- ☑ Active site
- ☑ Similarity

```
sp|ACA1_163470         448  TSARNLGPL-VSKRRGRDAP--------QRKSVDSGPATSESIALMAKSTSGLPTDDDDT   498
Q01484 ANK2_HUMAN     1000  APTRVTCRL-VKRHRLATMPPMVEGEGLASRLIEVGPSGAQFLGKLHL-----PT-----  1048
P06213 INSR_HUMAN      932  GNGSWTEPTYFYVTDYLDVPSN-------IAKIIIGPLIFVFLFSVVIGSIYLFLRKRQP   984
                                      .        *            :  **     :  :

sp|ACA1_163470         499  GGEDCNFEEVDAPEEREEDEAELSD-----SSGVEMKATIAVDSLSLDE----------H   543
Q01484 ANK2_HUMAN     1049  -----------APPPLNEGESLVSRILQLGPPGTKFLGPVIVEIPHFAAL-RG-KERELV  1095
P06213 INSR_HUMAN      985  DGPLGPLYASSNPEYLSAS-----D---------VFP--CSVYVPDEWEVSREKITLLRE  1028
                                        *   . .                :     *

sp|ACA1_163470         544  LGTGAFGDVYKGR------------LRGMTIV-------AVKKLTGVNQTAFLREAKIMQ   584
Q01484 ANK2_HUMAN     1096  VLRSENGDSWKEHFCDYTEDELNEILNGMDEVLDSPEDLEKKRICRIITRDFPQYFAVVS  1155
P06213 INSR_HUMAN     1029  LGQGSFGMVYEGNARDIIKGEAETRVAV-KTVNES--------ASLRERIEFLNEASVMK  1079
                            :  .  *  :: .            :     *                   * .   ::.

sp|ACA1_163470         585  QIPNHPNVVTFYGMAASQNVLYIVTEFVPHGSLALYLRSNIN---------KVPMHVLVQ   635
Q01484 ANK2_HUMAN     1156  RIKQDSNLIGPEGGVLSSTVVPQVQAVFPEGALTKRIRVGLQ---------AQPMHSELV  1206
P06213 INSR_HUMAN     1080  GFTCH-HVVRLLGVVSKGQPTLVVMELMAHGDLKSYLRSLRPEAENNPGRPPPTLQEMIQ  1138
                             :  . :::   * . .      *  ..  .* *   :*                ::  :

sp|ACA1_163470         636  MAKDIVSGMEHLRAN---DVVHRDLAARNLLLEI-RSNGEHRVKVSDFGLSRPLMESEYY   691
Q01484 ANK2_HUMAN     1207  --KKILGNKATFSPIVTLEPRRRKFH-KPITMTIPVPKASSDVMLNGFGGDAPTLRLLCS  1263
P06213 INSR_HUMAN     1139  MAAEIADGMAYLNAK---KFVHRDLAARNCMV-----AHDFTVKIGDFGMTRDIYETDYY  1190
                               .* ..   :      .  :*.:  :   :       .  * :..**      .

sp|ACA1_163470         692  KTGDSSRGHIPVKWTAPEALKWLKYSSQ---------SDVWAFGV----------APYPG   732
Q01484 ANK2_HUMAN     1264  ----ITGGTTPAQWEDITGTTPLTFVNECVSFTTNVSARFWLIDC----------RQIQE  1309
P06213 INSR_HUMAN     1191  RKG--GKGLLPVRWMAPESLKDGVFTTS---------SDMWSFGVVLWEITSLAEQPYQG  1239
                                   *  *.:*    . .   : ..         : .* :.

sp|ACA1_163470         733  LSNSEVIQEVSKGYRMPPPDNCPGQIVE--------------LMNECWHDDPL-------   771
Q01484 ANK2_HUMAN     1310  S-----VTFASQVYRE---IICVPYMAKFVVFAKSHDPIEARLRCFCMTDDKVDKTLEQQ  1361
P06213 INSR_HUMAN     1240  LSNEQVLKFVMDGGYLDQPDNCPERVTD--------------LMRMCWQFNPK-------  1278
                                  :  . .         *   :..              *   *   :

sp|ACA1_163470         772  ----------------KRPQWINIFVKLSALEE----KL---LDEWEEKERRREKDDREK   808
Q01484 ANK2_HUMAN     1362  ENFAEVARSRDVEVLEGKPIYVDCFGNLVPLTKSGQHHI---FSFFAFKENRLPLFVKVR  1418
P06213 INSR_HUMAN     1279  ----------------MRPTFLEI----VNLLKDDLHPSFPEVSFFHSEENKAPESEELE  1318
                                             :* :::       * :         .. :  :*.:     . .
```

**Figure 4.** Multiple sequence alignment of ACA1_163470 (top-row) human ankyrin repeat protein (middle-row) (ANK2_HUMAN Ankyrin-2) and human IR (bottom-row) showing regions of similarities, binding site, active site and transmembrane regions (colour legends top-left). **[Screenshots of BLASTp and MSA retrieved from Uniport (reference #16)]**

# Supplementary –File-Fig-5A

| | |
|---|---|
| Date of job execution | 2019-09-10 |
| Job identifier | A20190910674680338 1A1F0E0DB47453E0216320D0534879 (jobs are stored for 7 days) |
| Running time | 16.3 seconds |
| Identical positions | 370 |
| Identity | 35.957% |
| Similar positions | 305 |
| Program | CLUSTALO |

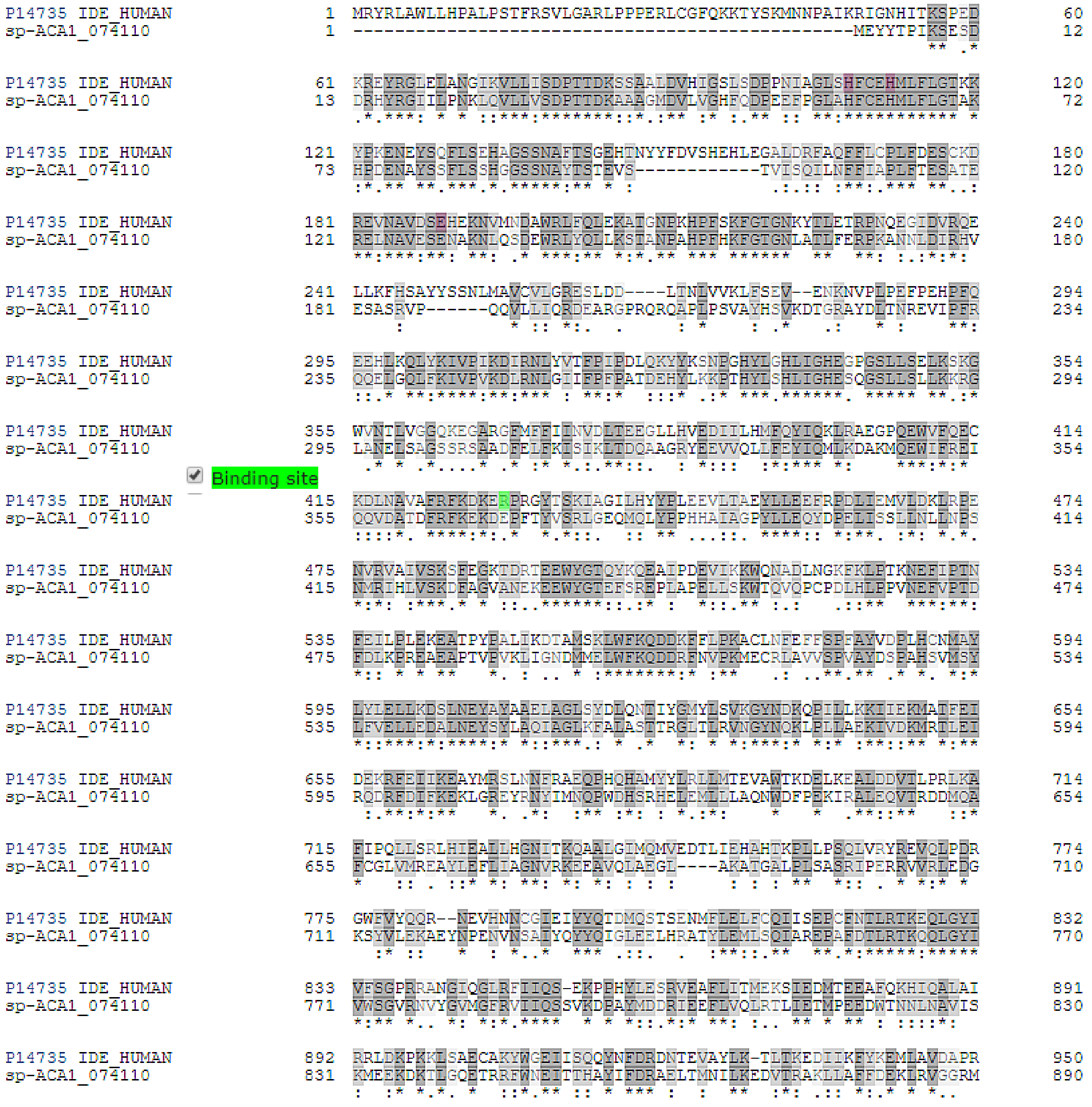


**Figure 5A.** BLASTp results show sequence between human insulin-degrading enzyme, to have a homolog ACA1_074110 in *A. castellanii* that had a high score, percentage of sequence identity and significant e-values. **[Screenshots of BLASTp and MSA retrieved from Uniport (reference #16)]**

# Supplementary File. Fig 5B

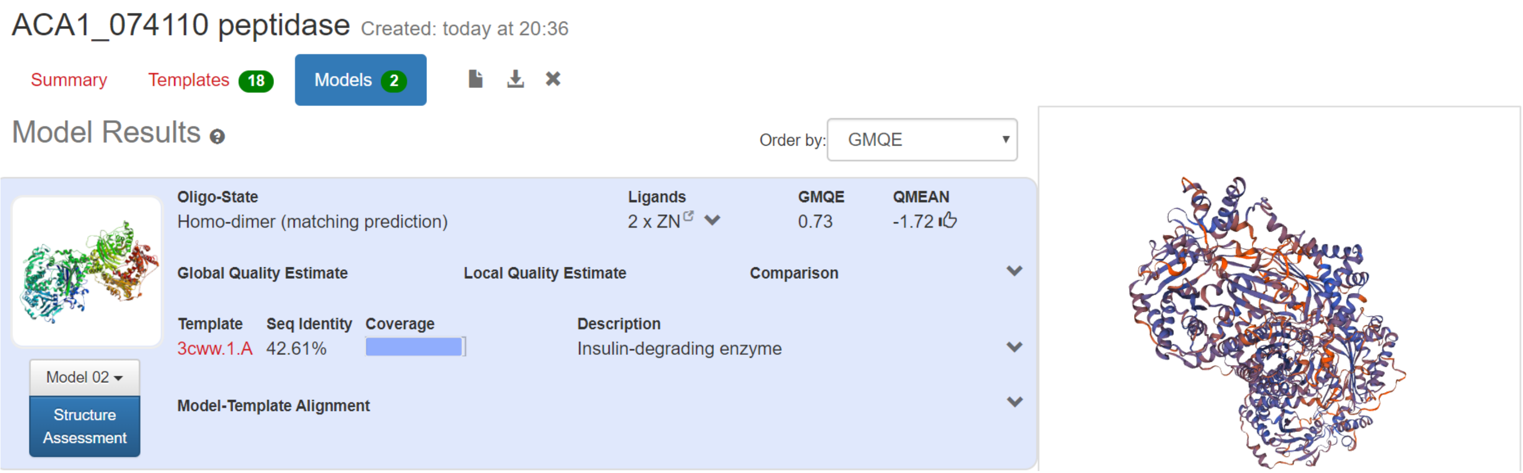


**Figure 5B:** Homology modelling of ACA1_074110 developed a template-based model of human Insulin degrading enzyme. **[Screenshots of structural model retrieved from SWISS-MODEL (reference # 24, 25)]**
